# Survivin/BIRC5-derived peptide disrupts survivin dimerization and cell division and induces multifaceted anti-cancer effects

**DOI:** 10.1016/j.omton.2025.201123

**Published:** 2026-01-03

**Authors:** Manikandan Santhanam, Venkatadri Babu, Anna Shteinfer-Kuzmine, Swaroop Kumar Pandey, Larisa Gheber, Gilead Raday, Varda Shoshan-Barmatz

**Affiliations:** 1 Department of Life Sciences, Ben-Gurion University of the Negev, Beer Sheva, Israel; 2 The National Institute for Biotechnology in the Negev, Ben-Gurion University of the Negev, Beer Sheva, Israel; 3 Department of Chemistry, Ben-Gurion University of the Negev, Beer Sheva, Israel; 4 RAD Therapeutics Inc, Westlake Village, CA, USA

**Keywords:** MT: Regular Issue, apoptosis, BIRC5/survivin, cancer, cell proliferation, peptides, tubulin, mitochondria, PD-L1, SMAC/Diablo

## Abstract

Survivin, a homodimeric protein overexpressed in virtually all cancers, is largely absent in non-proliferating adult tissues. It is a multifunctional regulator of cellular homeostasis that plays critical roles in proliferation, apoptosis, and immune regulation, which are central to cancer development and progression. Using a peptide array composed of sequences from SMAC/Diablo-interacting proteins, we identified a SMAC-binding sequence within survivin. Here, we report the characterization of a 24-amino-acid peptide spanning key survivin domains: the homodimer interface, microtubule, nuclear import, and chromosomal passenger complex binding sites. The peptide binds survivin and interferes with its dimerization, disrupting interactions with itself and partner proteins such as tubulin. When engineered as stabilized cell-penetrating peptides targeted to the cytosol, mitochondria, or nucleus, they effectively inhibited proliferation, disrupted the completion of mitosis, and induced apoptosis. In lung tumor models, the peptides reduced tumor cell proliferation and growth, while activating anti-tumor immune responses. They increased CD8^+^ T cell and NK cell infiltration and elevated PD-1/PD-L1 expression in the tumors. Additionally, they reduced the levels of survivin, SMAC, and tubulin, while increasing p53 expression in both *in vitro* and *in vivo* models. These findings highlight a novel strategy for targeting undruggable survivin using survivin-derived engineered peptides, offering promising therapeutic potential in cancer.

## Graphical abstract

**Figure:**
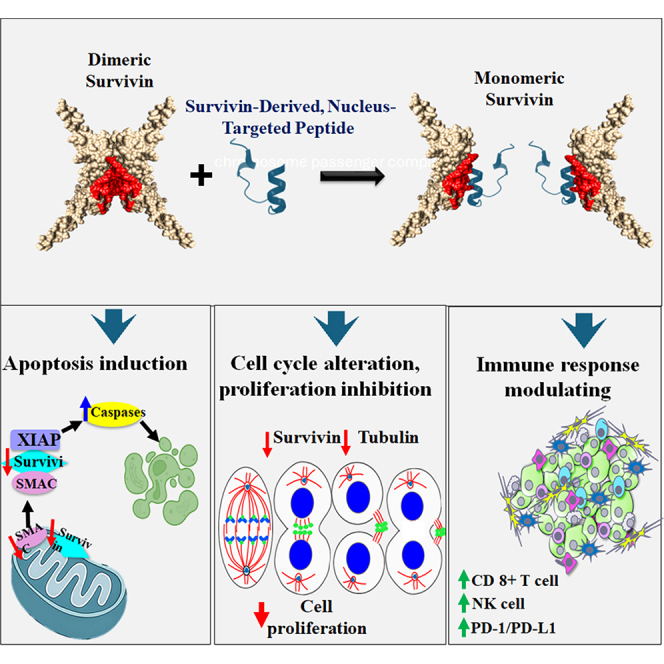

## Teaser

Survivin, an undruggable protein overexpressed in cancer, was targeted using a cell-penetrating peptide derived from its key functional domains. This peptide disrupts survivin dimerization and interactions with partners, inhibit cancer cell proliferation, impairs mitosis, and induces apoptosis. *In vivo*, it suppresses lung tumor growth and enhances anti-tumor immunity. Thus, offering a novel approach to cancer therapy.

## Introduction

Survivin, also known as baculoviral inhibitor of apoptosis repeat-containing 5 (BIRC5), is a member of the inhibitor of apoptosis (IAP) protein family that is able to bind caspases and prevent their activation, leading to apoptosis inhibition. Survivin is a multitasking protein that plays key roles in mitosis, apoptosis, autophagy, cell proliferation, cell division, regulation of the immune system, and mitochondrial integrity and function.^1^^,^^2^^,^^3^^,^^4^^,^^5^^,^^6^^,^^7^ It is found in the mitochondria, cytosol, nucleus,^8^^,^^9^ and exosomes.^10^ In tumors, survivin is localized in the mitochondria intermembrane space (IMS),^11^ where it suppresses apoptosis by binding SMAC/Diablo, a pro-apoptotic factor released from the mitochondria upon apoptosis activation,^12^ and that is essential for cancer cell proliferation and tumor growth.^13^^,^^14^^,^^15^ In the cytosol and nucleus, survivin regulates cell division by ensuring proper chromosome alignment, segregation, and kinetochore-microtubule attachment during mitosis and cytokinesis.^16^ It is a regulator of microtubule dynamics during cell division, ensuring correct kinetochore-microtubule attachment.^17^^,^^18^^,^^19^ Survivin also participates in regulating the immune system, such as in the development and differentiation of lymphocytes and dendritic cells (DCs).^20^^,^^21^

The survivin gene produces at least four alternatively spliced transcripts, each with unique roles in apoptosis, cell-cycle regulation, and therapy resistance.^1^^,^^22^^,^^23^^,^^24^ Wild-type survivin (survivin-1), its canonical form, consists of 142 amino acids and functions in inhibiting apoptosis and regulating cell division. Overexpression of wild-type survivin is observed in various cancers and is associated with poor prognosis, resistance to therapy, and increased tumor aggressiveness.^25^ Survivin-2B, containing an alternative exon 2, promotes proliferation, but has reduced anti-apoptotic function.^26^ Survivin-ΔEx3 lacks exon 3 and contributes to apoptosis and mitosis regulation but appears to be non-essential.^27^ Survivin-3B has an insertion of an alternative exon 3 and includes part of intron 2, possibly influencing cell division and apoptosis. This transcription varies across cancer types. Survivin-2α, derived from an alternative transcriptional start site, has pro-apoptotic activity and is expressed at low levels in tumors. Thus, the different isoforms of survivin are proposed to have distinct roles in apoptosis, cell division, and other cellular processes critical for cancer development and progression, and their expression levels vary according to cancer type and tumor stage.

Survivin is prominently expressed during embryonal development^28^ and is completely absent in terminally differentiated cells.^29^^,^^30^ In contrast, it is upregulated in many cancers, being the fourth most upregulated mRNA in the human cancer transcriptome.^25^^,^^31^^,^^32^^,^^33^^,^^34^^,^^35^^,^^36^^,^^37^ Its elevated expression correlates with poor clinical outcomes demonstrated in colorectal, breast, lung, glioma, and lymphoma cancers.^19^^,^^25^^,^^31^^,^^32^^,^^33^^,^^34^^,^^35^ In addition, apoptosis inhibition by survivin predicts a poor prognosis and shorter survival in patients suffering from carcinomas.^26^^,^^38^^,^^39^^,^^40^ These features make survivin a promising target for cancer therapies aimed at selectively perturbing tumor cell growth.

The active form of survivin is considered to be a homodimer of 16.5-kDa protein. Its 3D structure was resolved using X-ray crystallography and nuclear magnetic resonance, identifying the dimerization interface^41^^,^^42^ with the interacting residues between the two subunits including Leu6, Pro7, Pro8, Ala9, Trp10, Phe93, Glu94, Glu95, Leu96, Thr97, Leu98, Gly99, Phe101, and Leu102, with residues Leu98 and Phe101 critical for dimerization.^43^^,^^44^ Since the active survivin is a homodimer, disrupting the interacting interface through mutations or inhibitors impairs its activity and represents a promising cancer therapy strategy. In this study, we identified a survivin-derived sequence serving as an SMAC-binding site that also overlaps with its homodimerization sequence.

Survivin interacts with multiple proteins in numerous cellular pathways, contributing to its multiple roles in cancer development and progression, and other biological processes (Figure S1, Table S3). These interactions include: (a) the c*aspase family of proteins* that is involved in the activation cascade responsible for apoptosis execution. Survivin interacts with caspase-3, -7, and -9, at their catalytic site and/or at the dimerization site. This interaction blocks apoptosis execution and enables cancer cells to evade apoptosis^45^; (b) the *chromosomal passenger complex* (*CPC*): survivin is an essential component of the CPC, along with borealin, the aurora-B kinase, and INCENP. This complex ensures accurate chromosome alignment, centromere localization, spindle assembly checkpoint signaling, and successful cytokinesis during mitosis^46^; (c) *IAP family members*: survivin forms complexes with XIAP (X-linked inhibitor of apoptosis protein) c-IAP1 and BRUCE. These interactions contribute to the inhibition of caspases and promotion of cell survival.^47^ Complexes between survivin and XIAP have been reported; (d) *SMAC/Diablo*: survivin binds SMAC (second mitochondria-derived activator of caspases) or Diablo (direct IAP-binding protein with low pI) in an interaction that prevents caspase activation. Thus, inhibition of this interaction promotes apoptosis^48^^,^^49^^,^^50^; (e) *Beclin 1*: survivin interacts with Beclin 1, a central autophagy regulator that governs autophagosome formation and contributes to cellular homeostasis; (f) Bcl2 is a potent inhibitor of cell death that also acts as a regulator of the G2/M checkpoint and progression to cytokinesis during mitosis. Survivin has been shown to co-immunoprecipitate with Bcl-2 under specific conditions; (g) *cyclin-dependent kinases* (C*DKs*) are involved in the cell cycle. Interaction with CDKs regulates cell-cycle progression and mitosis.^51^ Survivin directly interacts with CDK1 and CDK4, and CDK1 directly phosphorylates survivin on threonine 34 during mitosis; (h) *signal transducer and activator of transcription 3* (*STAT3*): survivin interacts with STAT3, which can modulate its expression and functions in cancer cells^52^; (i) *tubulin* (*microtubules*): During mitosis, survivin binds to tubulin/microtubules and localizes to the mitotic spindle, contributing to proper mitotic progression.^2^^,^^53^ Survivin also binds to microtubules in interphase cells, but the role of this interaction is unclear; (j) *non-muscle myosin II* (*NMII*): survivin binds NMII, regulating its filament assembly. This interaction is essential for successful cytokinesis^54^ (Table S3); (k) *phosphatidylserine decarboxylase* (*PSD*) interacts with survivin and inhibits its activity^13^; (l) *MYC*, a proto-oncogene and transcription factor, forms a complex with survivin, as demonstrated by co-immunoprecipitation. In addition, MYC enhances survivin expression by binding to its promoter, while survivin, in turn, protects MYC from proteasomal.

Other non-direct interactions have also been reported: Wild-type p53 suppresses survivin expression, while mutant p53 leads to its upregulation, contributing to therapy resistance.^55^ Similarly, CD40, a type I transmembrane protein, interacts with its ligand to induce survivin expression in both immune and cancer cells, thereby promoting cell survival and resistance to apoptosis.^56^

The previous interactions position survivin as a central player in regulating apoptosis, cell cycle, mitosis, and oncogenic transformation, making a compelling target for cancer therapy. Because survivin overexpression in cancer promotes cell proliferation and inhibits apoptosis—critical processes in cancer development and progression^2^^,^^3^^,^^4^^,^^5^^,^^6^^,^^7^—multiple therapeutic strategies have been developed to reduce its expression or suppress its activity.^43^^,^^57^^,^^58^^,^^59^^,^^60^^,^^61^^,^^62^^,^^63^^,^^64^^,^^65^ These include blocking its transcription and destabilizing the protein or inhibiting its interactions with other proteins. Small molecules like YM155,^65^ RNA-based methods (e.g., siRNAs, shRNAs),^62^ and CRISPR-Cas9^63^ have been used to reduce survivin expression. Several miRNAs (e.g., miR-16, miR-34a, miR-143, miR-150, miR-203, miR-494, and miR-708) have also been shown to downregulate survivin.^60^^,^^61^ Additionally, immunotherapies, such as cancer vaccines and checkpoint inhibitors that aim to enhance immune responses against survivin-expressing cells have also been explored.^64^ However, despite these efforts, survivin remains largely undruggable, as discussed further in the Discussion section.

Recently,^66^ we identified the SMAC-binding site on survivin, and based on the sequence of this site, we developed stabilized cell-penetrating peptides targeted to either the nucleus or mitochondria. These peptides showed strong anti-tumor activity, inhibiting tumor growth, reducing inflammation, and alleviating immunosuppression in pre-clinical models.^66^

In the current study, we focused on a nucleus-targeted survivin-derived peptide to explore this peptide mechanism of action. The peptide binds to survivin and alters its dimerization and interaction with tubulin. As a cell-penetrating peptide, in cells in culture and lung cancer mouse model, it reduced the expression of survivin, SMAC, and tubulin, and disrupted cell division and triggered apoptosis. It also enhanced CD8^+^ T cell and natural killer (NK)-cell infiltration into tumors and upregulated PD-1/PD-L1 expression, demonstrating its combined impact on tumor cell function and the immune microenvironment.

## Results

### Survivin-derived peptide binds to survivin and modulates its dimerization

Using the STRING database (Figure S1A), about 32 survivin-interacting proteins were proposed. However, the resulting network includes not only direct physical interactions between survivin and other proteins, but also indirect associations such as shared pathway involvement or regulatory effects on survivin expression. Survivin expression is unregulated by p53,^55^^,^^67^ whereas caspase-2 represses its transcription, and silencing caspase-2 expression upregulates its expression.^68^ As we are interested in proteins that directly interact with survivin, we present only those that have been experimentally validated (Figure S1B, Table S3). These proteins are found in mitochondria, cytoplasm, nucleus, and plasma membrane and are involved in key processes such as inflammation, ubiquitination, apoptosis, cell-cycle regulation, and proliferation (Table S3). Selected survivin-interacting proteins include caspases (2, 3, 7–9); BIRC2 and BIRC6; Bcl-Xl; XIAP; JAK1, 2, and 3; STAT3; CDKs (1, 8, 9); CD-40; PSD; non-muscle myosin,^54^ tubulin,^2^^,^^53^ and SMAC/Diablo.^48^^,^^49^^,^^50^ Survivin’s interaction with these proteins establishes its broad functional role in cancer-related pathways.^69^

**Figure 1:**
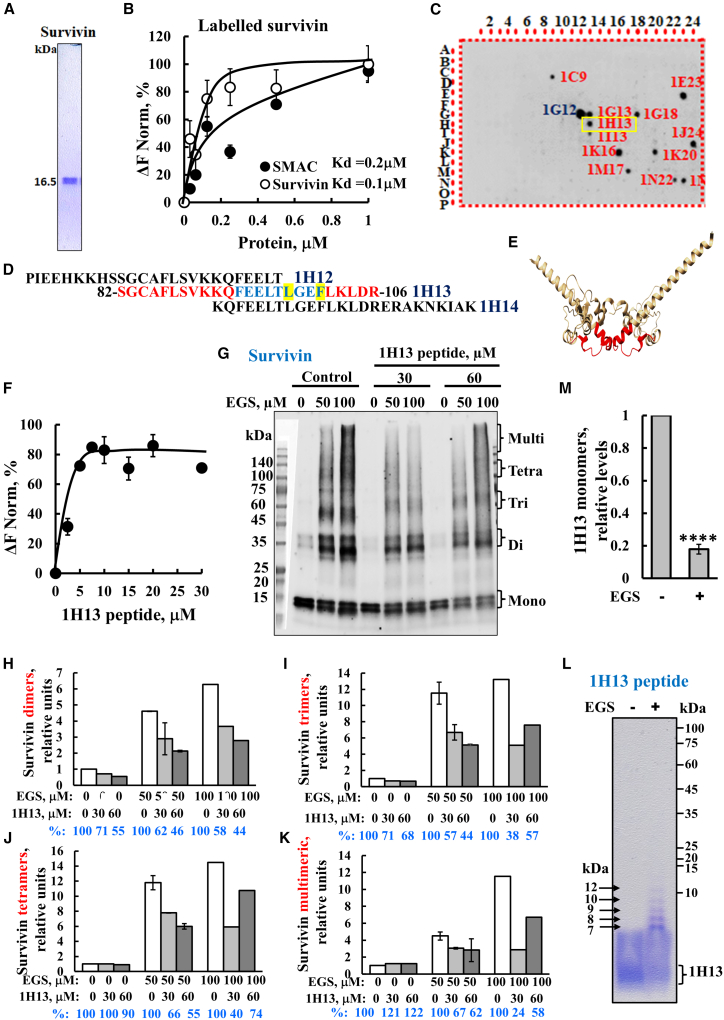
Interaction of survivin with SMAC, identification of the binding site that as peptide interferes with survivin assembly into dimers and higher oligomeric states (A) Purified survivin used in this study was purchased from Sino Biological (Beijing, China, Cat. SINO-10356HNCE). (B) Survivin binding to itself and to SMAC. Purified survivin was fluorescently labeled as described in the materials and methods. Kd for survivin and SMAC are 100 and 200 nM, respectively. (C) Identifying the SMAC binding site in survivin. A glass-bound peptide array consisting of overlapping peptides derived from 11 selected SMAC-interacting proteins was incubated overnight with purified SMAC (0.8 μM) and then blotted with anti-SMAC antibodies (1:1000), followed by incubation with HRP-conjugated secondary antibodies and detection using a chemiluminescence kit. Dark spots indicate the binding of SMAC to peptides derived from SMAC-interacting proteins, such as with spots 1G12 and 1E23, derived from BIRC2 and 1H13 derived from survivin. A representative blot of three independent experiments is shown. (D) Overlapping sequences of peptides derived from BIRC5 in the glass-bound peptide array. The sequence of the peptide that interacted with SMAC is in red, and the peptides located before and after appearing in black. The peptide localization in dimeric survivin in each subunit of the homodimers with the proposed stabilized interacting residues Phe93, Glu94, Glu95, Leu96, Thr97, Leu98, Gly99, Phe101, and Leu102, which were all present in the peptide (in blue, and Leu98 and Phe101 highlighted in yellow). (E) Dimeric survivin crystal structure, (PDB_1e31), in the red the peptide location the image was prepared using UCSF Chimera.^70^ (F) Survivin interaction with the indicated concentrations of the survivin 1H13-derived peptide analyzed using the MST method. Fluorescently labeled purified survivin (650 nM) was incubated for 30 min at 37°C with the peptide (2–30 μM), and thermophoresis was measured as described in (B). Kd = 2 ± 0.05 μM (*n* = 3). (G) Purified survivin (1 μg/mL) was incubated in the absence and presence of an 1H13 peptide (30 or 60 μM) in PBS, pH 8.3 (15 min, 30^o^C), and then incubated with EGS (50 or 100 μM, 15 min, 30^o^C). The reaction was terminated by adding sample buffer, followed by SDS-PAGE (gradient gel 4%–20% acrylamide), followed by immunoblotting with anti-survivin antibodies The positions of the survivin monomers, dimers, trimers, tetramers, and higher oligomers are indicated. (H–K) The levels of survivin dimers, trimers, tetramers, and multimers were quantified using ImageJ software and presented relative to their levels in the EGS-untreated samples and their levels in the presence of the peptide compared to its absence (shown in blue). (L and M). IH13 peptide (60 μM) in PBS, pH 8.3 was incubated with EGS (50 μM, 15 min, 30^o^C), followed by SDS-PAGE (gradient gel 4%–20% acrylamide) and Coomassie blue staining. The levels of peptide dimers, trimers, tetramers, and multimers are indicated (L). Peptide levels (monomeric) before and after crosslinking (M).

Given the significance of survivin homodimerization and of the SMAC-survivin interaction, we validated these using purified proteins and microscale thermophoresis (MST). Fluorescently labeled survivin was incubated with increasing concentrations of either SMAC or survivin (Figures 1A and 1B). The results demonstrate the direct interactions of survivin with SMAC and with itself, with a relatively high affinity with a dissociation constant (Kd) of ∼200 and ∼100 nM, respectively.

To identify SMAC-interacting sequences within survivin, we used a peptide array composed of 768 peptides derived from 11 selected SMAC-interacting proteins, among them survivin/BIRC5 (Figure 1C). The array was incubated with purified SMAC, followed by anti-SMAC antibodies and then with HRP-conjected secondary antibodies, and developed by ECL (electrogeneated chemiluminescence). The dark spots represent SMAC-binding peptides (Figure 1C). Among the survivin-derived peptides, a single spot was detected (peptide 1H13), representing binding to SMAC. This 25-amino-acid peptide overlaps by 15 amino acids with adjacent peptides 1H12 and 1H14, which did not bind SMAC despite sharing ∼64% sequence identity (Figure 1D). The location of the 1H13 within the survivin sequence (Figure S2A) and in the available 3D structures for the survivin/BIRC5 dimer (Figure 1E) are shown. 1H13 lies within the survivin dimerization interface sequence and includes the SMAC-binding site (Figure 1E), as it was identified in the peptide array (Figure 1C).

The direct interaction of the synthetic 1H13 peptide with purified survivin was demonstrated using the MST method, yielding a dissociation constant (Kd) of ∼2 μM (Figure 1F).

Survivin dimerization is mediated by specific residues at the interface between its two identical subunits, including Leu6, Pro7, Pro8, Ala9, Trp10, Phe93, Glu94, Glu95, Leu96, Thr97, Leu98, Gly99, Phe101, and Leu102, with Leu98 and Phe101 being critical for dimer formation.^41^^,^^42^^,^^43^^,^^44^ The 1H13 peptide sequence, spanning amino acids 83–106, encompasses this dimerization region, including the essential Leu98 and Phe101 residues.

To assess the peptide’s effect on survivin dimerization, purified survivin was incubated with or without the 1H13 peptide and then subjected to crosslinking using the crosslinker EGS, followed by immunoblotting (Figures 1G–1K). Interestingly, upon crosslinking, survivin showed not only dimers, but also trimers, tetramers, and higher-order multimers (Figure 1G). The levels of monomeric, dimeric, trimeric, and oligomeric forms of survivin were quantified (Figures 1H–1K). At the tested concentrations, the 1H13 peptide reduced survivin oligomer formation by up to 60% (presented at the bottom of Figures 1H–1K), suggesting that it interferes with survivin self-association. In this respect, dynamic light scattering (DLS) experiments have revealed that all tested survivin preparations (1 mg/mL) exhibited significant physical heterogeneity, containing species from monomers and dimers to large complexes, and that in the presence of EDTA, extremely large aggregates formed (∼400Å in diameter, i.e., 106 kDa).^71^

Since the peptide includes the dimerization domain, we next examined whether it could self-associate to form dimers and other oligomeric forms. For this purpose, the peptide was subjected to crosslinking with EGS and then analyzed by gel electrophoresis and Coomassie blue staining. Results showed a decrease in the monomeric peptide due to its crosslinking (Figures 1L and 1M) and an increase in several forms of dimers (7 kDa and 8 kDa), trimers (9 kDa and 10 kDa), and other higher oligomeric states (12 kDa) (Figure S2C). The appearance of several dimer and trimer forms may result from crosslinking by EGS of different lysine residues in the two peptides (Figures S2B–S2C).

### Survivin-derived peptides, targeted to cytosol, nucleus, or mitochondria induce apoptosis

**Figure 2:**
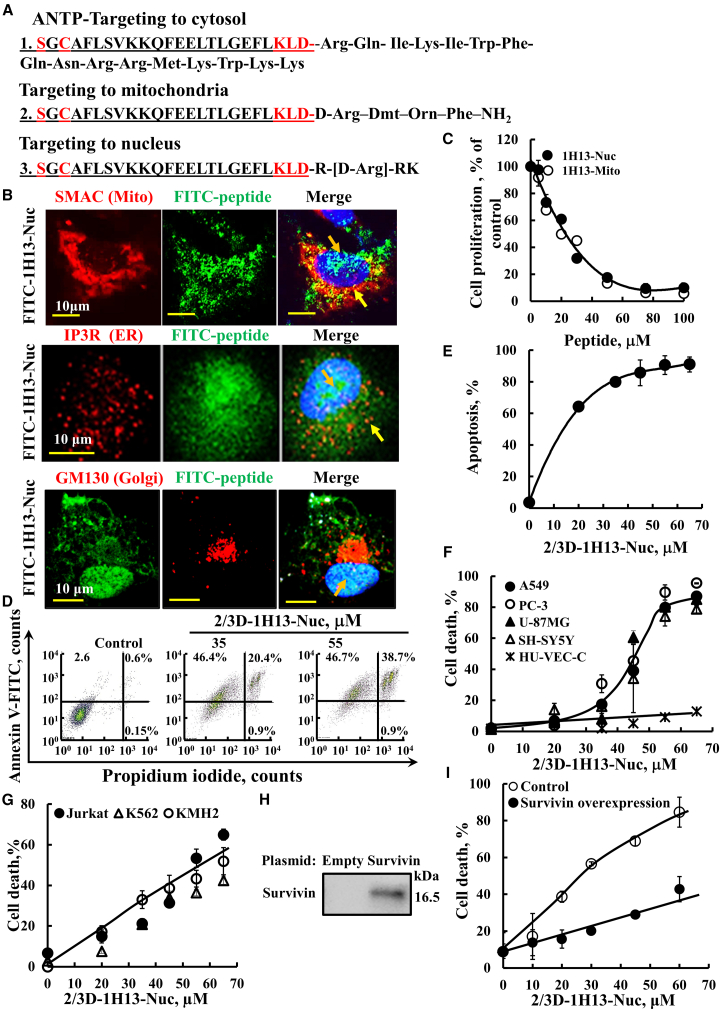
Cell death induction by 1H13-survivin/BIRC5-derived peptides targeted to the cytoplasm, mitochondria, or nucleus (A) The sequences added to the 1H13-BIRC5 peptide to target it to the: (1) cytoplasm, (2) mitochondria, or (3) nucleus. The underlined sequence represents the 1H13 peptide; in red, amino acids indicate D-amino acid substitutions. (B) A549 cells were incubated for 90 min with 5 μM FITC-labeled, nucleus-targeted peptide IH13-Nuc, and were also IF-stained with anti--SMAC, anti-IP3R or anti-GM130 antibodies, and stained with DAPI to visualize the mitochondria, ER, Golgi, and nucleus, respectively. Confocal microscope images are shown, with white arrows indicating peptide co-localization with the mitochondria (SMAC). Orange and yellow arrows indicate peptide presence in the nucleus and cytosol, respectively. (C) A549 cells were incubated with the mitochondria- or nucleus-targeted 1H13-BIRC5-derived peptide for 24 h in serum-free medium, followed by a cell proliferation assay using the SRB method. (D and E) Apoptotic cell death as induced in A549 cells following incubation for 24 h with the nucleus-targeted peptide (2/3D-1H13-Nuc) in the presence or absence of the indicated concentrations of the peptides in serum-free medium and subjected to FITC–annexin V/PI staining, followed by a flow cytometry analysis. Representative histograms for control and selected peptide concentration (D) and analysis of early and late apoptotic stages are shown (E). (F and G) Cell death as induced by 2/3D-1H13-Nuc in different cell lines, A549, SH-SY5Y, U-87MG, PC-3, and HUV-EC-C (F) or Jurkat, K562, and KMH2-LC (G) were treated with the indicated concentrations of the peptide for 24 h, then subjected to cell death analysis using propidium iodide (PI) staining and flow cytometry. (H and I) A549 cells were seeded at a density of 2 × 10^5^ cells per well in a 12-well plate. After 24 h, the cells were transfected with 2 μg of a pCMV3-survivin expression plasmid (HG10356-UT, Sino Biological, China) or with an empty pCMV3 plasmid (control) using JetPrime transfection reagent (Polyplus, France), following the manufacturer’s instructions. Twenty-four hours post-transfection, the cells were re-seeded at 1 × 10^5^ cells per well in a 12-well plate. After another 24 h, the culture medium was replaced with serum-free medium, and the cells were treated with the indicated concentrations of the 2/3D-1H13-Nuc peptide. Survivin overexpression levels were assessed by immunoblotting (H), and cell death was analyzed by propidium iodide (PI) staining followed by FACS analysis (I). Results represent the means ± SEM (*n* = 3).

Survivin has been shown to be located in the cytosol, mitochondria, and the nucleus.^8^^,^^9^ Accordingly, we previously developed a survivin/BIRC5-derived peptide (1H13) in cell-penetrating forms (CPPs) targeted to the cytosol, mitochondria, or nucleus, and demonstrated their ability to induce cell death.^66^ In this study, we designed additional CPP forms of the 1H13 peptide with substitutions of L-amino acids with their D enantiomers to enhance stability (Figure 2A). Cytoplasmic targeting was achieved by adding to the peptide cell-penetrating sequence (Antp/penetrating) derived from the *Drosophila* antennapedia homeodomain.^72^ For mitochondrial targeting, the peptide was fused to a mitochondria-targeting sequence composed of D-Arg–Dmt–Orn–Phe–NH_2_,^73^ while for nuclear targeting, the peptide was fused to the tetrapeptide RrRK (r = D-arginine).^74^

Previously, we demonstrated targeting of the specific sequence to the right compartment.^66^ Here, we analyzed the cellular distribution of the nuclear targeted survivin/BIRC5-derived 1H13 peptide (1H13-Nuc) by labeling it with FITC and assessing the FITC-labeled peptide distribution in A549 cells using confocal microscopy (Figure 2B). We found that this peptide was localized primarily in the nucleus (co-localized with DAPI), but that it was also detected in the cytosol and mitochondria, where it was co-localized with SMAC (mitochondria) (Figure 2B).

The possible localization of the 1H13-Nuc peptide to the endoplasmic reticulum (ER) and/or Golgi apparatus was examined by analyzing the co-localization of the FITC-labeled peptide with IP3R and GM130, markers of the ER and Golgi, respectively (Figure 2B). Partial co-localization of the peptide with IP3R was observed, while no overlap with GM130 was detected. Given the close physical association between the ER and mitochondria at the mitochondria-associated membranes (MAMs), the resolution of confocal microscopy does not allow us to clearly distinguish whether the peptide resides within the ER or at mitochondria-associated ER membranes.

Both peptides targeted the nucleus (1H13-Nuc) or the mitochondria (1H13-Mito) strongly inhibited cell proliferation (Figure 2C).

In addition, we tested cell death induction by peptides targeted to the cytosol, mitochondria, and nucleus, where selected L-amino acids were replaced with the corresponding D-enantiomers. Modifications included either all the D-amino acids (All-D-1H13) or only two and three at the N- and C-terminius, respectively (2/3D-1H13). The cytosol targeted peptides, all L-1H13-Antp, 2/3D-1H13-Antp, and all-D-1H13-Antp induced maximal cell death of 90%, 90%, and 30% (at 30 μM), respectively (Figures S3A–S3C). The cell death induction of the various peptide forms targeted to the mitochondria or nucleus is expressed as IC_50_ (concentration for 50% cell death) and maximal cell death (Figure S3C). The 2/3D-1H13-peptides exhibited activity similar to or less than the full L-amino acid version. In contrast, all-D-1H13 peptides showed reduced affinity and lower maximal cell death (Figure S3C).

The 2/3D-1H13-Nuc peptide induced apoptosis, as confirmed by Annexin V–FITC and propidium iodide (PI) staining, followed by flow cytometry analysis (Figures 2D and 2E). Early apoptotic cells were reduced, while late apoptotic cells increased in a peptide concentration-dependent manner.

The effect of the peptide was also evaluated across several cell lines (Figures 2F and 2G). The results indicate that the adherent cancer cell lines A549, PC-3, U-87MG, and SH-SY5Y displayed similar sensitivity to the peptide, with half-maximal cell death observed at approximately 45 μM. In contrast, the non-cancerous HUV-EC-C cell line showed no signs of cell death under the same conditions. The peptide also induced cell death in suspension cell lines; including Jurkat, K562, and KMH2 (Figure 2G). Survivin expression levels and its subcellular localization were examined in the adherent cancer cell lines A549, PC-3, HeLa, and SH-SY5Y using anti-survivin and anti-ATPsyn5a (mitochondrial marker) antibodies, with DAPI staining for the nucleus (Figure S4). Survivin levels were comparable across all cell lines and localized to both mitochondria and the nucleus, consistent with their similar sensitivity to peptide-induced cell death (Figures 2F and 2G).

The relationship between survivin expression levels and the peptide’s cell death-inducing activity was further examined by transfecting cells with either a control plasmid or a plasmid expressing survivin (Figures 2H and 2I). The results show that in cells with elevated survivin levels, the concentration of peptide required to induce 50% cell death increased approximately 3-fold.

### Survivin-derived 2/3D-1H13-Nuc peptide reduces survivin, SMAC, and Ki-67 expression, while increasing p53 levels

**Figure 3:**
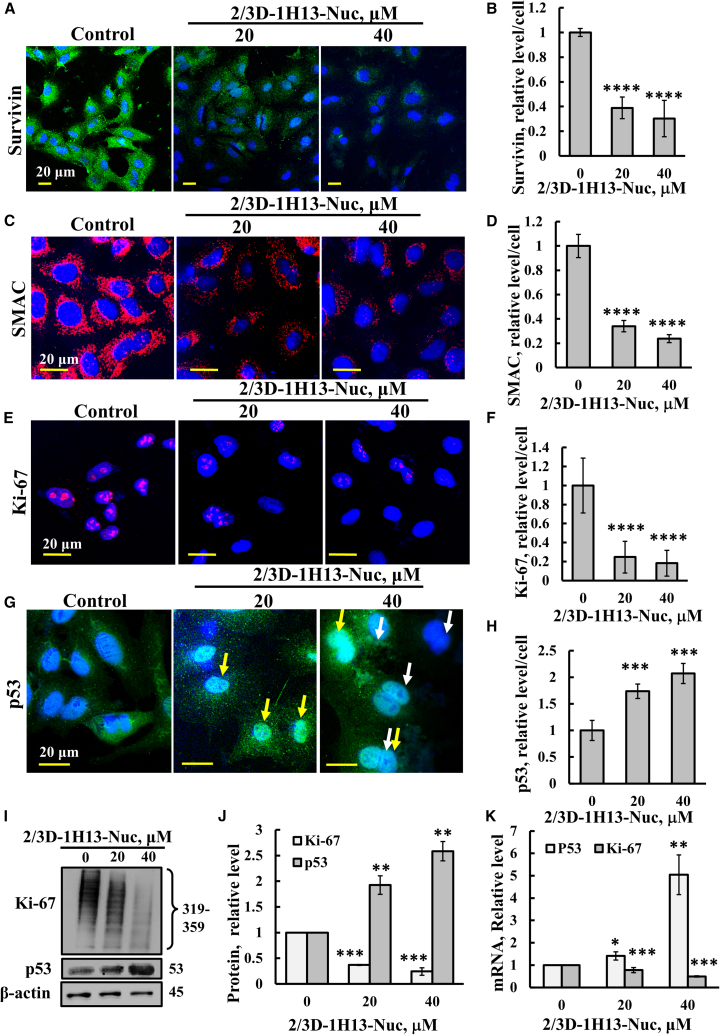
2/3D-1H13-Nuc decreases survivin, SMAC, and Ki-67 levels, and increases p53 levels A549 cells were seeded on 13-mm glass coverslips, untreated (control) or treated with the 2/3D-1H13-Nuc peptide (20 or 40 μM, 24 h), fixed and subjected to IF using anti-survivin (A and B), anti-SMAC (C and D), anti-Ki-67 (E and F), or anti-p53 antibodies (G and H). The yellow and white arrows point to nuclear p53 and binuclear cells, respectively (G). Confocal microscope images are shown (A, C, E, and G), and quantification of the staining intensity per/cell (120–140 cells analyzed for each sample) is presented (B, D, F, and H). (I and J) Cells treated with the 2/3D-1H13-Nuc peptide for 24 h, harvested, and then subjected to immunoblotting using specific antibodies against p53, Ki-67, or β-actin (I), and band intensities were quantified using ImageJ software (J), or subjected to RT-qPCR for quantification of mRNA levels of p53, Ki-67, or β-actin (K), as described in the Method section. Results represent the means ± SEM (*n* = 3); ∗∗*p* ≤ 0.01; ∗∗∗*p* ≤ 0.001; ∗∗∗∗*p* ≤ 0.0001.

We observed a significant reduction in survivin (Figures 3A and 3B) and SMAC (Figures 3C and 3D) expression in cells treated with the 2/3D-1H13-Nuc peptide, as assessed by immunofluorescence using specific antibodies. Additionally, the peptide markedly decreased the expression of the proliferation marker, Ki-67, as analyzed using specific antibodies (Figures 3E and 3F). In contrast, p53 expression in peptide-treated cells was increased, along with its nuclear translocation (Figures 3G and 3H). This agrees with our previous findings that showed that the 2/3D-1H13-Nuc peptide suppresses survivin expression and leads to increased caspase-3 and activation of apoptosis, mitotic deregulation, and increased sensitivity to anti-cancer drugs.^75^^,^^76^ The peptide ability to decrease Ki-67 and increase p53 expression levels was confirmed by immunoblotting (Figures 3I and 3J) and by RT-qPCR (Figure 3K). The RT-qPCR results showed reduced mRNA level of Ki-67 and elevated mRNA levels of p53, suggesting that the peptide exerts its effects at the transcription level.

### Survivin interacts with tubulin and the survivin-derived 2/3D-1H13-Nuc peptide reduces tubulin expression and modulates the cell cycle

**Figure 4:**
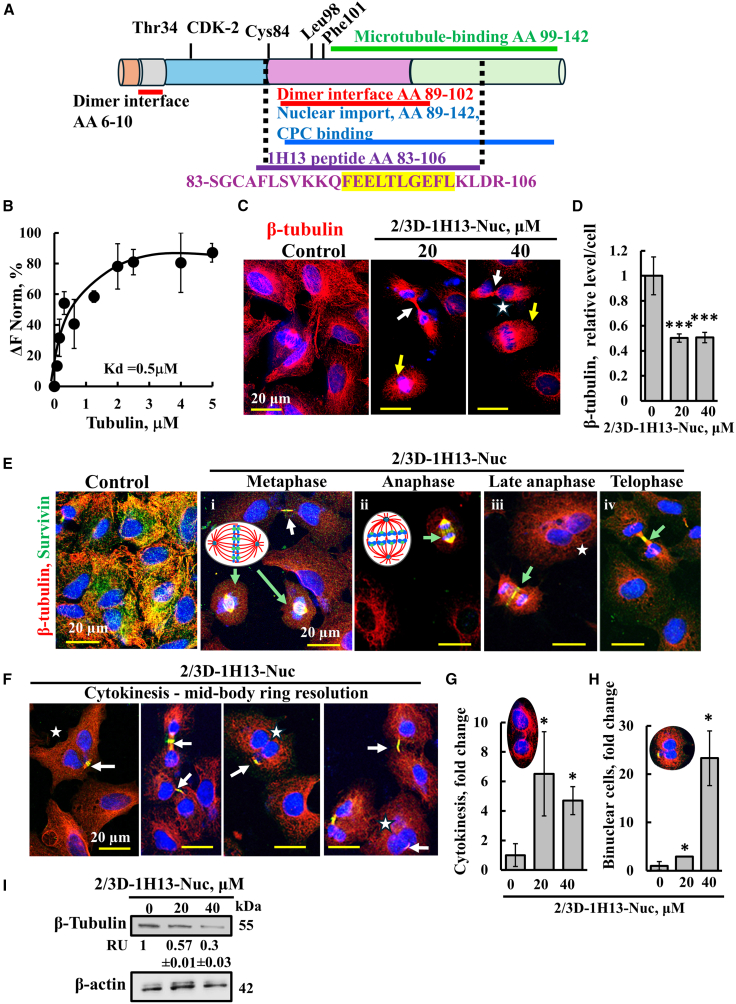
Survivin colocalizes to microtubules and the 1H13-Nuc peptide decreases tubulin levels and impairs cell division (A) Schematic representation of survivin amino acid sequence indicating the following structural features: dimerization domains (AA 6–10 and 89–102, red lines), tubulin binding domain (AA, 99–142, green line) chromosomal passenger complex (CPC) binding, nuclear import domains (AA 89–142, blue line) and the peptide sequence and its location (AA 83–106, in purple) . The overlapping sequence between the dimerization, tubulin, the CPC and the peptide 1H13 is labeled by dashed lines. Threonine 34 undergoing phosphorylation, the CDK-2 binding site, mitochondria targeting (MTS, arrowhead), Lue98 and Phe101, required for dimerization, and the microtubule binding domain. (B) MST analysis of tubulin binding to survivin was performed using a NanoTemper Monolith NT.115 apparatus (See Materials and Methods and legend of Figure 1). Kd survivin-tubulin binding = 500 nM. (C and D) A549 cells were seeded on 13-mm glass coverslips, untreated (control) or treated with the 2/3D-1H13 peptide (20 or 40 μM, 24 h), fixed, and subjected to IF using anti-β-tubulin antibodies. Confocal microscope images are shown, with white arrows indicating cells in cytokinesis and yellow arrows indicate cell division (C). Quantification of tubulin levels per cell was analyzed in the IF-stained sample using ImageJ (120–140 cells were analyzed for each sample) (D). (E and F) A549 cells were seeded on 13-mm glass coverslips, untreated (control) or treated with the 2/3D-1H13-Nuc peptide (40 μM, 24 h), fixed, and subjected to co-IF using anti-β-tubulin and anti-survivin antibodies. Confocal images demonstrate the co-localization of tubulin and survivin (control) and in the peptide-treated cells (E and F), cell division stages: metaphase, anaphase, telophase, indicated by green arrows and on top, white arrow point to cytokinesis (E); cytokinesis and binuclear cells are shown (F). In C, E, and F, asterisk indicates binuclear cells. The quantified proportion of cells undergoing cytokinesis (G) and binuclear cells (H) in peptide-treated cells are shown relative to their levels in untreated cells, indicating a significant increase of cytokinesis and binuclear cells upon peptide treatment. (I) Cells treated with the indicated concentration of 2/3D-1H13-Nuc peptide for 24 h, harvested, and then subjected to immunoblotting using anti-tubulin or anti-β-actin antibodies, and band intensities were quantified using ImageJ software and presented as relative units (RU). Results represent means ± SEM (*n* = 3); ∗*p* ≤ 0.05; ∗∗∗*p* < 0.001.

The structural features of survivin also include, in addition to the dimerization domain (amino acids, 1–6 and 89–102, Figure 4A; red line), a chromosomal passenger complex (CPC) binding domain and nuclear import sequence (amino acids 89–142, Figure 4A; blue line), and a microtubule-binding site sequence (amino acids 99–142, Figure 4A; green line). The 1H13 peptide sequence represents amino acids 83–106 and overlaps with the dimerization domain and part of the microtubule binding, CPC binding, and nuclear import domains (Figure 4A, violet line). Consistent with the 1H13 peptide including part of the microtubule-binding domain of survivin, we found that purified survivin bound to purified β-tubulin with relatively high affinity (Kd = 500 nM) (Figure 4B). We also found that the treatment of cells with the 2/3D-1H13-Nuc peptide reduced tubulin levels in the cells (Figures 4C and 4D). The peptide-induced decrease in tubulin expression is confirmed by immunoblotting (Figure 4I).

It has been previously demonstrated that survivin is essential for proper cell division, and its loss leads to the disruption of the mitotic checkpoint, leading to polyploidy and cell death.^77^^,^^78^^,^^79^ It was shown to play a critical role in cytokinesis, and to be essential for targeting the CPC to the midbody, in addition to targeting the CPC to the centromeres and central spindle during earlier stages of mitosis.^80^

Thus, the impact of the peptide on mitosis and the cell cycle was further examined through IF- staining for both survivin and tubulin (Figures 4C, 4E–4H, S5, and S6). In control cells, both proteins were highly expressed and co-localized (Figure 4E). In peptide-treated cells, the levels of both survivin and tubulin were markedly reduced, and the phases of mitosis—including metaphase, anaphase, and telophase—were clearly visible (Figure 4Ei-iv). Indeed, our results show that cell division was impaired in the presence of the peptide. In addition to reducing cell numbers due to inhibited proliferation (Figure 2C) and induced apoptosis (Figures 2D–2G), in the presence of the peptide, various phases of mitosis were found (see below), with a majority of cells being arrested in cytokinesis (Figures 4C–4H).

During metaphase, when the chromosomes align along the equatorial plate of the microtubule-based mitotic spindle, survivin was co-localized with the spindle microtubules (Figure 4Ei). In anaphase, sister chromatids were pulled toward opposite poles, remaining attached to the spindle microtubules by their centromeres, with survivin and microtubules co-localizing as the chromatids moved toward the poles (Figures 4Eii and 4Eiii). During telophase, the furrow formed an intercellular bridge made of mitotic spindle fibers, with survivin visible within the furrow (Figure 4Eiv). The cleavage of the furrow was driven by an actin-myosin contractile ring, which tightened around the cytoplasm, physically separating the daughter cells.^81^ Upon peptide treatment, the majority of the cells were found at this stage, indicating failure to proceed with full cell division (Figures 4F and 4G), resulting in incomplete cytokinesis.

Moreover, we observed that following treatment with high peptide concentration, there was an accumulation of binuclear cells (Figures 4E, 4F, and 4H), further indicating failure to complete cytokinesis. This effect was dependent on functional p53 in the A549 cells,^82^ since in the PC-3 cells which lack functional p53,^83^ no accumulation of cells in cytokinesis and/or binuclear cells was observed (Figure S6). Instead, following peptide treatment, the size of the nuclei in the PC-3 cells was significantly larger compared to controls (Figure S6C), indicating progression to the next G1 stage, without completing mitotic DNA division, likely resulting in aneuploidy.^77^

Thus, in the presence of the peptide, survivin was detected at the kinetochores, cleavage furrow, and midbody, all of which are associated with microtubules during metaphase to cytokinesis. It caused an accumulation of cells with double or large nuclei (depending on the p53 status), which highlights the critical role of survivin in ensuring the successful completion of mitosis and maintaining genomic stability.

### Survivin/BIRC5-derived 1H13 peptides inhibited tumor growth and induced apoptosis in a lung cancer xenograft model

**Figure 5:**
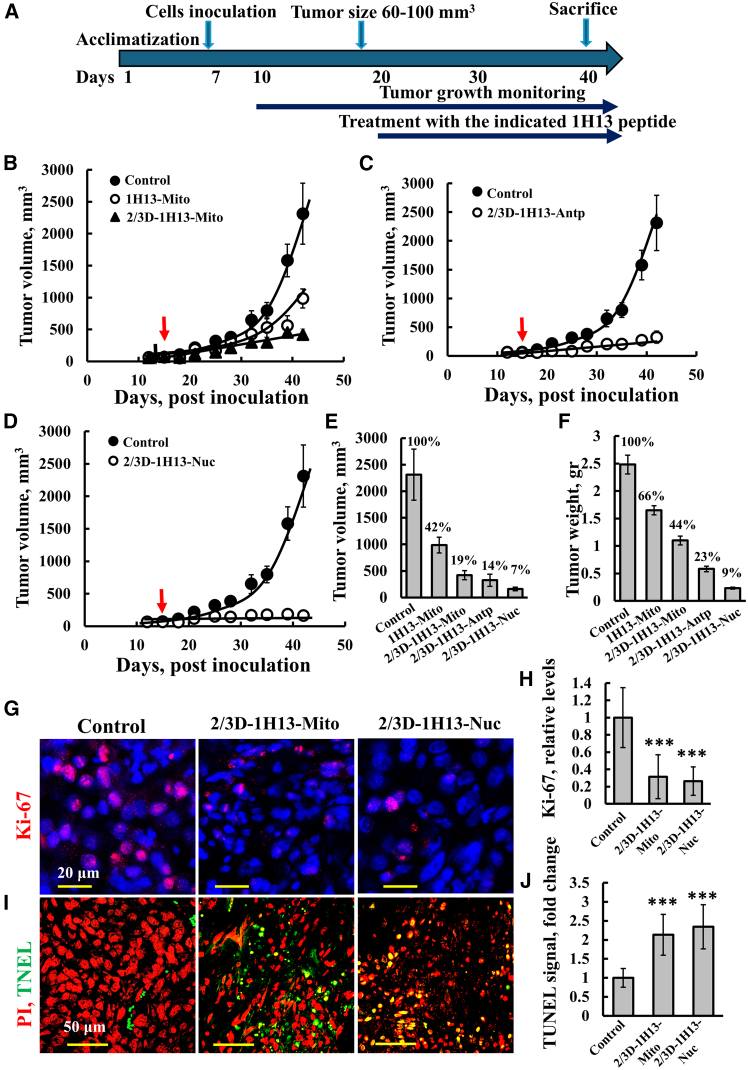
2/3D-1H13 peptide targeted to the nucleus, mitochondria, or cytosol, inhibits tumor growth of A549 lung cancer xenografts (A) Experimental protocol: Lung adenocarcinoma A549 cells (7 × 10^6^ cells/mouse) were inoculated subcutaneously (s.c.) into female nude mice. The tumor sizes were measured (using a digital caliper), and the volume was calculated. When the tumor volume reached 60–100 mm^3^ (day 15), the mice were divided into several groups: control and treated, with the indicated peptide (20 mg/kg, i.v. twice a week). (B, C, and D) Tumor volume of untreated (control) and treated mice with mitochondria targeted peptides (1H13-Mito or 2/3D-1H13-Mito) (B), or with cytosolic targeted peptides (2/3D-1H13- Antp) (C), or with nuclear targeted peptides (2/3D-1H13- Nuc). (E) Average tumor volume of all groups at day 42. (F) After 42 days, the tumors from the various groups were dissected and weighed. (G–J). Sections of control and tumor-treated mice with the indicated 1H13-peptide were IF-stained with specific antibodies against Ki-67 (G) or TUNEL stained (I), and quantified (H and J) to analyze cell proliferation and cell death, respectively. Results represent means ± SEM; ∗∗∗*p* < 0.001.

We treated established A549 lung cancer xenografts in nude mice with an intravenous (i.v.) injection of the 1H13-BIRC5 peptide targeted to the cytosol, mitochondria, or nucleus, with and without D-amino acid modifications at the N- and C-terminus (2/3D-1H13-BIRC5) (Figure 5). As outlined in the experimental protocol (Figure 5A), peptide treatment began when the tumors reached an approximately volume of 100 mm^3^ and was administered twice per week over a four-week period. In the control group, tumors grew exponentially, increasing approximately 40-fold over 42 days. In contrast, all of the peptide-treated tumors exhibited substantial growth inhibition (Figures 5B–5E). After 42 days, the tumor volume in the peptide-treated mice was significantly reduced, with the D-amino acid-modified mitochondrial-targeted peptide reducing tumor volume by about 58%, compared to 81% for the all-L-amino acid peptide (Figure 5E). The nuclear-targeted peptide, 2/3D-1H13-Nuc, proved to be the most effective, inhibiting more than 90% of tumor growth. Similar results were observed when tumor weight was monitored, with an over 90% reduction in the mice treated with 2/3D-1H13-Nuc (Figure 5F).

Peptide-treated tumors also showed a 90% decrease in Ki-67 levels, a marker of cell proliferation, as analyzed by immunostaining (Figures 5G and 5H). Additionally, TUNEL staining revealed significant DNA fragmentation, indicating increased cell death (Figures 5I and 5J).

**Figure 6:**
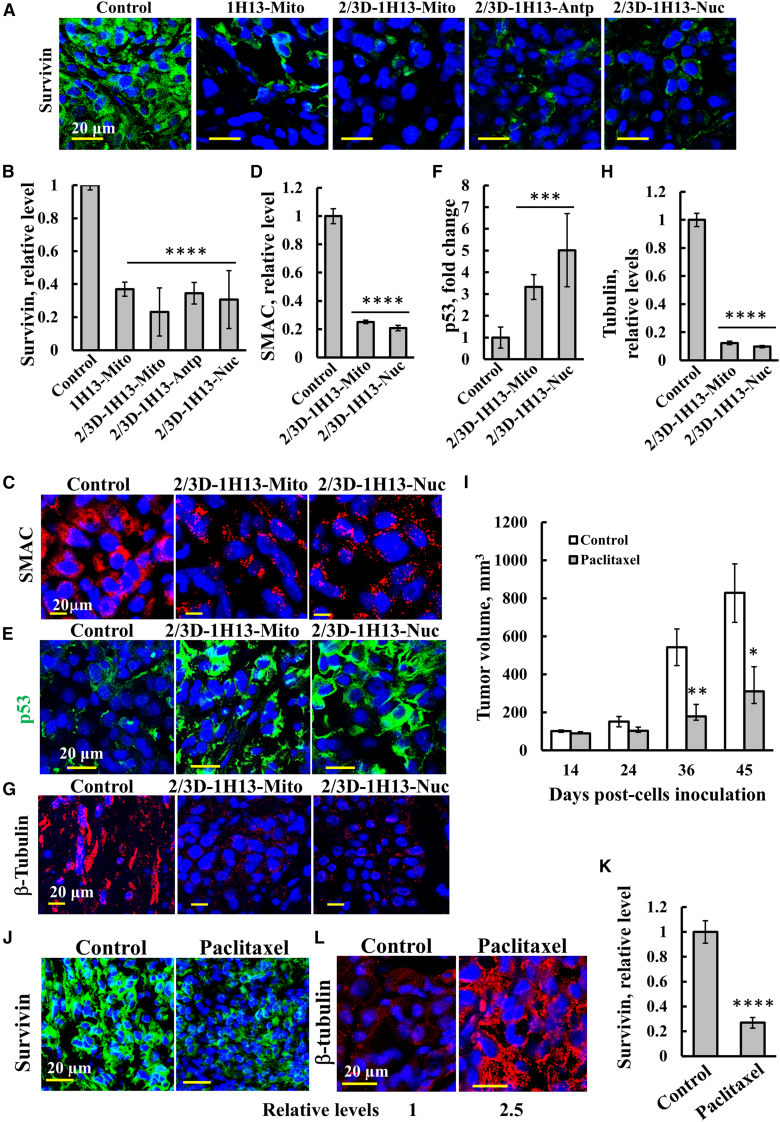
1H13 peptides targeted to the nucleus, mitochondria, or cytosol inhibit decreased expression of survivin, SMAC, p53, and β-tubulin in tumors Sections of control and tumor-treated mice with the indicated 1H13-peptide were IF-stained with specific antibodies for survivin (A and B), SMAC (C and D), p53 (E and F) or β-tubulin (G and H). Representative confocal images are shown (A, C, E, and G), and staining intensity quantification from three different tumors each (B, D, F, and H) are shown. (I–L) Paclitaxel treatment of a breast cancer xenograft mouse model inhibits tumor growth, stabilizes microtubules, and reduces survivin expression. (I) Athymic female nude mice were s.c. injected with MDA-MB-231 (3×10^6^), and tumor formation was followed. Upon reaching a volume of 50–100 mm^3^ (day 14), the mice were split into two tumor volume-matched groups and were i.v. injected three times a week with the vehicle (NaCl 0.9%) or with paclitaxel (10 mg/kg), followed by measurement of the tumor volume. Tumor volume as a function of time post-cell inoculation is presented. (J–L) 45 days post-cell inoculation, the tumors were dissected, fixed, and embedded in paraffin. Sections of control and paclitaxel-treated tumors were IF-stained for survivin (J and K) or β-tubulin (L) using specific antibodies, and staining intensities were quantified (K) or indicated in the bottom of the image (L). Results represent the means ± SEM (*n* = 3), ∗*p* ≤ 0.05; ∗∗*p* ≤ 0.01; ∗∗∗∗*p* < 0.0001.

We next analyzed the expression of survivin, SMAC, p53, and tubulin in the tumors of both groups untreated and peptide-treated mice (Figure 6). Tumors from the mice treated with any of the four 1H13-BIRC5 peptides targeted to the cytosol, mitochondria, or nucleus with or without D-amino acid modification (2/3D-1H13-BIRC5) showed significant reductions in survivin (Figures 6A and 6B) and SMAC (Figures 6C and 6D) levels, while p53 expression was markedly increased, particularly by the nuclear-directed peptide (Figures 6E and 6F). Tubulin levels were significantly reduced by approximately 90% in the peptide-treated tumors (Figures 6G and 6H), consistent with the results observed in cultured cells (Figures 3 and 4).

### Paclitaxel effects on tumor progression may be associated with reduced survivin levels

Paclitaxel is a microtubule-stabilizing agent commonly used in the treatment of various cancers, inducing mitotic arrest and cell death.^84^^,^^85^ Thus, we assessed the impact of paclitaxel (Taxol) on survivin expression.

In our study, paclitaxel treatment significantly inhibited the growth of MDA-MB-231 breast cancer cell-derived tumors, reducing tumor size by approximately 75% (Figure 6I). IF-staining for survivin revealed a marked reduction in survivin expression in tumors from paclitaxel-treated mice (Figures 6J and 6K). Conversely, β-tubulin levels were notably increased in the same tumors (Figure 6L), likely due to paclitaxel’s stabilizing effect on microtubules.^84^ This reduction in survivin levels may contribute to the therapeutic effects of paclitaxel.

### Survivin-derived peptides targeted to the mitochondria or the nucleus modulate tumor immunity

**Figure 7:**
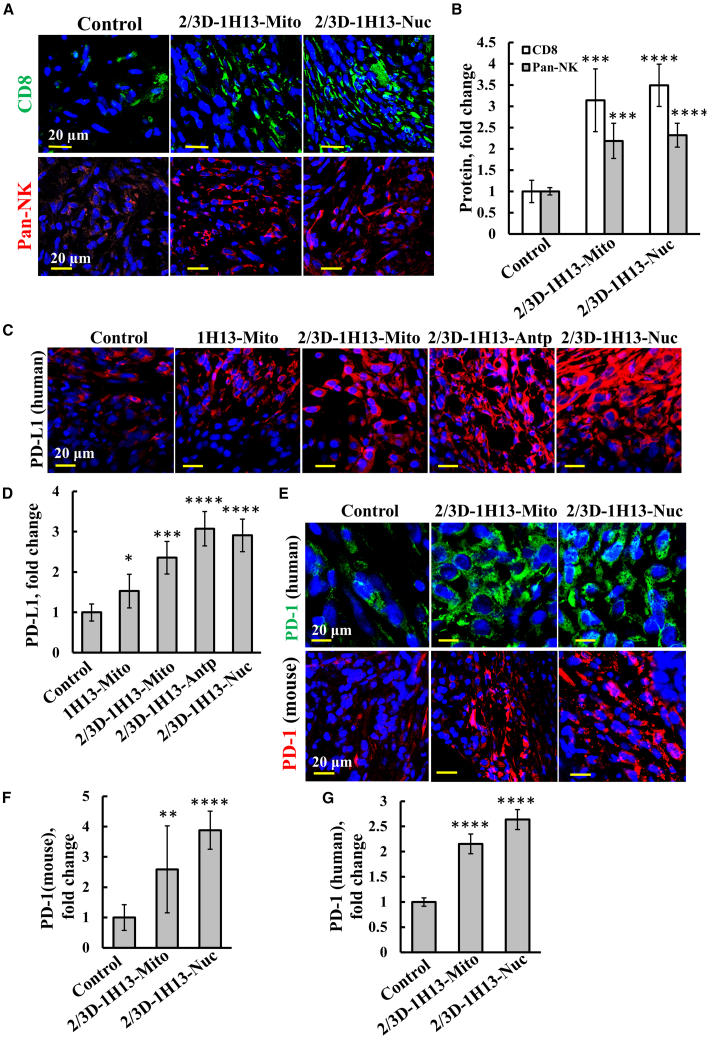
2/3D-1H13 peptide targeted to the cytosol, mitochondria, or nucleus induced infiltration of CD8^+^ T and NK cells into the tumor and increased PD-L1 expression in cancer cells (A and B) Sections of tumors from control and mice treated with the 2/3D-H13 peptide targeted to the mitochondria or nucleus were IF-stained for CD8 or NK cells using specific antibodies (A) and staining intensity quantification (B). (C and D) Tumor sections from control and treated mice with 2/3D-H13 peptide targeted to mitochondria, cytosol, or the nucleus were IF-stained with anti-PD-L1 (human) (C), and the staining intensity was quantified (D). IF-staining for mouse and human PD-1, using specific antibodies (E), and their staining intensity quantification (F and G). Results represent mean ± SEM (*n* = 3). ∗*p* ≤ 0.05; ∗∗*p* ≤ 0.01;∗∗∗*p* ≤ 0.001; ∗∗∗∗*p* < 0.0001.

Tumor-infiltrating immune cells include activated T cells, NK cells, B lymphocytes, macrophages, dendritic cells (DCs), and monocytes.^86^ CD8 (cluster of differentiation 8) is a cell surface glycoprotein found on most cytotoxic T lymphocytes, that their infiltration into solid tumors is often associated with a favorable prognosis in certain cancers.^87^ Here, using IF with specific antibodies, we demonstrated that treatment with the 2/3D-1H13-Nuc or 2/3D-1H13-Mito peptides led to a significant increase in CD8^+^ T cells within the tumor (Figures 7A and 7B). Similarly, NK cell levels were also notably elevated in tumors from the peptide-treated mice (Figures 7A and 7B).

Programmed cell death protein 1 (PD-1) and programmed cell death ligand 1 (PD-L1) are critical regulators of T cell activation, proliferation, and cytotoxic secretion, impeding the anti-tumor immune response.^88^ Tumor cells can escape immune surveillance by overexpressing PD-L1, which binds to the PD-1 expressed on immune cells, promoting their death, and thereby preventing an immune response.^89^ Treatment with 1H13-peptides, targeted to the mitochondria (L or 2/3D modified), cytosol, or nucleus, resulted in significantly increased expression of human PD-L1 in tumors (Figures 7C and 7D).

Peptide-treated mice also showed a strong upregulation of PD-1 in non-cancerous cells, as revealed by anti-PD-1 staining using mouse-specific antibodies (Figures 7E and 7F). Furthermore, as we demonstrated previously,^66^ IF-staining with human-specific anti-PD-1 antibodies revealed that tumors from peptide-treated mice exhibit high levels of PD-1 expression in cancer cells (Figures 7E–7G). This is consistent with prior studies showing that PD-1 is expressed in non-small lung cancer cells (NSCLC) patient samples and cell lines such as A549, used in this study.^90^ Notably, our findings indicate not only that the peptides induced the expression of both PD-L1 and PD-1 in the same cancer cell, but also in two subpopulations: some cells expressed PD-1, while others expressed PD-L1 (Figure S7).^66^ In cancer cells, ligation of PD-1 to its ligand, PD-L1, can induce apoptosis, cell-cycle arrest, and anergy.^90^^,^^91^ PD-L1 can also suppress antitumor immunity primarily by inactivating CD8^+^ T cells.^92^

## Discussion

Survivin is highly expressed in most cancer types, with elevated levels linked to tumor aggressiveness and resistance to chemotherapy.^25^^,^^93^^,^^94^ In contrast, it is rarely present in non-proliferative adult tissues and only transiently expressed in proliferating cells.^29^ For instance, survivin is overexpressed in ∼86% of lung cancer cases.^25^ Its widespread expression and multifunctional role in cancer make survivin an appealing therapeutic target. However, due to its lack of enzymatic activity, most strategies have focused on disrupting survivin interactions, blocking homodimerization, inhibiting gene transcription, targeting mRNA, or using survivin-based immunotherapies.^43^^,^^57^^,^^58^^,^^59^^,^^60^^,^^61^^,^^62^^,^^63^^,^^64^ Despite extensive research, translating these strategies into clinical success has proven challenging.^43^^,^^57^^,^^58^^,^^59^

**Figure 8:**
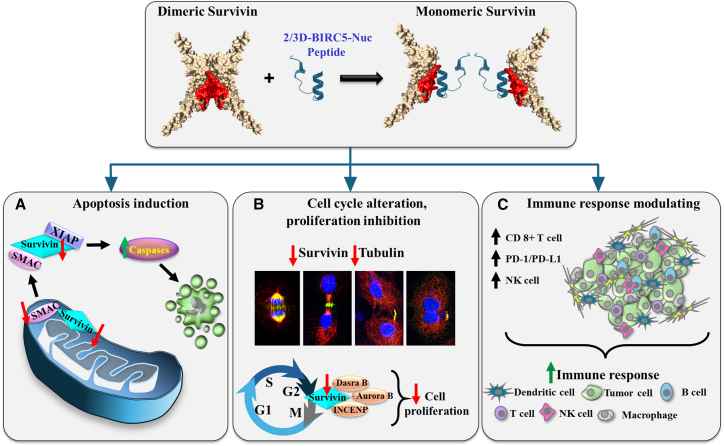
Proposed mechanisms for the multiple effects of the survivin-derived peptide in tumors: a possible new multi-target treatment for cancer schematic of the mechanism of action of the multiple effects of the survivin/BIRC5-derived peptide on cancer hallmarks: Survivin/BIRC5 dimer interacting with the peptide leading to dissociation of the dimer. This leads to (A) Apoptosis—survivin binding to SMAC prevents caspase activation, and thereby apoptosis, (B) Altered cell cycle due to decreased tubulin levels and/or checkpoint activation—survivin being part of the CPC and directs the aurora-B kinase to the centromere during mitosis, thereby inhibiting cell proliferation, and (C) Modulating the immune response—increasing the tumor infiltration of CD-8 and activated NK cells and increasing the expression of PD-1 and PD-L1 on cancer cell sub-populations results in their interaction, and leads to cancer cell death, while sparing the immune cells.

The major findings of this study are summarized in Figure 8. Here, we developed a survivin-derived peptide containing its key dimerization sequence. This peptide binds survivin and modulates its dimerization and oligomerization. When targeted to the cytosol, mitochondria, or especially the nucleus, the peptide reduces the levels of survivin, SMAC, and tubulin, while increasing p53.

It also induces apoptosis, disrupts cell division, and inhibits proliferation. In a lung cancer mouse model, the peptide significantly suppressed tumor growth through multiple mechanisms, including direct survivin inhibition, and enhanced tumor immunity. This multifunctional agent represents a novel and promising approach to targeting survivin in cancer therapy.

### Survivin-derived peptide modulates survivin dimerization state

Survivin forms homodimers with its 3D structure, as resolved using X-ray crystallography and NMR spectroscopy, identifying specific interactions at the dimerization interface,^41^^,^^42^ with the amino acid residues spanning 94–99 in survivin form antiparallel β-sheet in the dimerization interface.^42^ The homodimer is stabilized by hydrophobic residues Leu6, Pro7, Pro8, Ala9, Trp10, Phe93, Glu94, Glu95, Leu96, Thr97, Leu98, Gly99, Phe101, and Leu102, with the core interaction involving Phe93, Leu98, and Phe101, making hydrophobic contacts with their counterpart residues on the other monomer.^43^^,^^95^ Computational studies also highlight Leu98 and Phe101 as core interaction residues.^44^

Using a peptide array of 768 peptides from SMAC-interacting proteins, we identified the survivin-derived peptide (1H13, Ser83 to Arg106), representing the proposed dimerization sequence and including the critical dimerization residues Leu98 and Phe101^42^^,^^43^^,^^44^^,^^95^ (Figure 1D). Moreover, the survivin sequences that overlapped with this sequence, containing 64% identical sequencing (IH12 and 1H14), do not bind to SMAC, suggesting that additional residues like Leu87 are also crucial for SMAC interaction.^96^ Chemical crosslinking showed that survivin forms not only dimers, but also higher-order oligomers, including trimers, tetramers, and multimers (Figure 1). Oligomeric survivin has been demonstrated to form dimers and very large aggregates up to 106 kDa.^71^ In addition, aggregation of survivin around histone H3 EMs has been demonstrated and proposed to point to its potential regulatory function in gene transcription.^97^ The peptide decreased the formation of all oligomeric forms of survivin, suggesting it competes with survivin for binding and disrupts survivin oligomerization (Figure 1G). Moreover, the peptide, which represents the dimerization interface, also undergoes oligomerization (Figure 1L).

Disrupting survivin dimerization has been proposed as a potential approach to interfere with its oncogenic function. *In-silico* screening of 200,000 compounds identified a small molecule that targets the key dimerization residues, Leu98 and Phe101.^44^ However, as discussed in the section *Survivin as a target to treat cancer*, no approved therapies currently target survivin directly. This study demonstrates that a peptide derived from the survivin dimerization interface can target otherwise undruggable homodimeric proteins. As summarized in Figure 8, this peptide disrupts the multiple functions of survivin, affecting cell-cycle regulation, inhibiting cell proliferation, inducing apoptosis, modulating the immune response, and altering the expression of proteins in survivin’s network that are involved in cancer progression. These effects can explain the dramatic effect of the peptide on tumor growth.

### Survivin-derived peptides targeted to cytosol, mitochondria, and most prominently, the nucleus induce cell death, reduce survivin and SMAC, but increase p53 levels

We show that survivin/BIRC5-derived peptides, targeted to the cytosol, mitochondria, and especially the nucleus, inhibit cell proliferation and induce apoptosis *in vitro* and *in vivo* (Figure 2, Figure 3, Figure 4, Figure 5, Figure 6, Figure 7 and S3). Survivin is localized in the cytoplasm, mitochondrial intermembrane space, and nucleus,^8^^,^^9^ (Figure S4). The peptide targeted to these compartments activated apoptosis in both cultured cells and tumors (Figures 2, and 5, S3). Nuclear-, cytosolic-, and mitochondria-targeted 1H13 peptides elicited similar apoptotic responses, likely reflecting survivin’s dynamic localization among cellular compartments. These findings suggest that organelle-targeted peptides can interfere with both cytoplasmic and organelle-associated survivin functions, consistent with survivin’s multifunctional presence in the cytoplasm, nucleus, and mitochondria.

To improve stability and reduce immunogenicity, L-amino acids were substituted with D-amino acids, yielding an active peptide effective in a lung cancer xenograft model administered intravenously.

Survivin/BIRC5, by interacting with SMAC, functions as anti-apoptotic protein.^98^

As survivin is often overexpressed in cancer and linked to progression and therapy resistance,^43^^,^^57^^,^^58^^,^^59^^,^^60^^,^^61^^,^^62^^,^^63^^,^^64^^,^^65^ it represents a promising therapeutic target.

Survivin has a short half-life of 30 min and is degraded by the proteasome upon polyubiquitination. Our peptides significantly reduced survivin levels in both cultured cells (Figures 3A and 3B) and tumors (Figures 6A and 6B). It has been proposed that exposure of the hydrophobic interface of a dimeric protein causes destabilization and degradation through the proteasome or autophagy.^99^ By interfering with survivin dimerization, the peptide potentially promotes the degradation of survivin.

Survivin overexpression in cancer cells disrupts microtubule dynamics and cell division, promoting tumorigenesis and chemoresistance.^100^ We found that paclitaxel, a microtubule-stabilizing agent, significantly reduces survivin levels in tumors (Figures 6J and 6K). While short-term Taxol treatment increases survivin in MCF-7 cells, longer treatment (48 h) decreases its expression.^101^ In ovarian carcinoma patients, survivin overexpression was linked to poor response to Taxol/platinum regimens, but not cisplatin alone, suggesting that paclitaxel’s efficacy may involve survivin downregulation.^102^ Given survivin’s roles in cell division, apoptosis, and inflammation, some paclitaxel effects may stem from its reduction.

In previous studies, we showed that SMAC is essential for cancer cell survival. Reducing its levels with siRNA or CRISPR-Cas9 decreased cell proliferation phospholipid transport and synthesis, metabolism; altered epigenetics, cell signaling, and neutrophil-mediated immunity; and reduced survivin expression.^13^^,^^14^^,^^15^ In this study, we found that the survivin-derived peptide reduced SMAC levels in both cultured cells and tumors (Figures 3C and 3D, 6C, and 6D), mimicking SMAC knockdown and further supporting the role of SMAC expression in survivin’s anti-apoptotic activity.^13^^,^^14^^,^^15^

The tumor suppressor p53 regulates the cell cycle and induces apoptosis. It binds directly to the survivin promoter, suppressing its transcription and promoting cell-cycle arrest.^55^ Survivin overexpression inhibits p53-dependent apoptosis. In our study, peptide treatment increased p53 levels in both cultured cells and tumors (Figures 3G and 3H, 6E, and 6F), likely due to reduced survivin. Additionally, prior studies also show crosstalk between p53 and survivin at both transcriptional and protein levels,^103^ and that survivin inhibition can activate the p53 pathway and sensitize cancer cells to poly ADP-ribose polymerase inhibition.^104^

How the survivin-derived peptide alters the expression of multiple proteins remains unclear. The peptide may influence transcription factors that regulate gene expression, mimicking survivin’s reported roles in gene expression. It is found in both the nucleus and cytoplasm of cancer cells,^3^^,^^8^ and recent studies suggest that it interacts with the nuclear export receptor Crm1, influencing cancer-relevant functions of survivin.^105^^,^^106^ Additionally, survivin can bind Dicer promoter, suppress its transcription, and reduce global miRNA expression,^107^^,^^108^ potentially contributing to its broad effects on protein expression.

### Survivin-derived peptide decreased tubulin expression, altered cell division, and inhibited cell proliferation

Survivin stabilizes microtubules by binding tubulin through its dimeric form and stabilizes tubulin via a microtubule-binding domain (amino acids 99–142).^17^^,^^19^ This binding site overlaps with the 1H13 peptide (aa 83–106) (Figure 4A). Direct binding between tubulin and purified survivin was confirmed (Figure 4B). Targeting the peptide to the nucleus or mitochondria significantly reduced tubulin expression in both cells and tumors (Figures 4 and 6, and S5), while paclitaxel increased tubulin levels (Figure 6L), consistent with reports of paclitaxel-induced ßIII-tubulin overexpression in chemically induced rat mammary tumors.^109^

Survivin is essential for cell division, ensuring proper mitotic spindle function and chromosome segregation, and it is indispensable for cytokinesis,^110^ a function associated with its interaction with tubulin.^18^ Our results suggest that the peptide disrupts survivin-tubulin interactions, impairing cell division and contributing to reduced proliferation (Figures 2C and 4C, 4E–4H, and S5).

The survivin sequence also contains nuclear import domains and a chromosomal passenger complex (CPC) binding site (aa 89–142)^18^^,^^80^ (Figure 4A). As part of the CPC, along with aurora kinase B and others, survivin ensures proper chromosome segregation and cytokinesis by its localizing to mitotic structures such as centrosomes, kinetochores, and midbodies^8^^,^^16^^,^^18^^,^^80^^,^^111^^,^^112^, and it is involved in forming the contractile ring during cytokinesis,^54^ playing a direct role in cytokinesis.^110^

Treatment with the nucleus-targeted survivin-derived peptide (2/3/D-1H13-Nuc) reduced survivin levels and caused cytokinesis failure, resulting in either bi-nucleated cells (Figures 4F–4H) or cells with large nuclei in PC-3 cells not expressing p53 (Figures S6A–S6C). Similar defects are seen with survivin depletion, including spindle loss, aberrant mitosis, and checkpoint failure.^18^^,^^80^

Disrupting survivin-tubulin interactions with the peptide impaired cell division and induced cell death, supporting its potential as a cancer therapy.^43^^,^^57^^,^^58^^,^^59^^,^^60^^,^^61^^,^^62^^,^^63^^,^^64^^,^^65^ Targeting survivin may also enhance sensitivity to microtubule-targeting drugs like taxanes.

### 2/3D-1H13-survivin/BIRC5-derived peptide treatment modulates tumor immunity

Survivin is implicated in the pathogenesis of inflammatory and autoimmune disorders,^113^ with dysregulated expression detected in various autoimmune diseases.^114^^,^^115^^,^^116^^,^^117^^,^^118^ It also influences remodeling of the tumor microenvironment by mediating reciprocal signaling among immune cells, tumor cells and stromal niche components.^119^ Previously,^66^ we showed that the survivin-derived peptide increased CD8^+^ T cell infiltration. Here, we demonstrate that it also enhances NK cell infiltration (Figures 7A and 7B).

CD8^+^ T cells play a key role in anti-tumor immunity, and their increased infiltration predicts a positive response to immunotherapies^87^ and NK cells contribute innate immune defense without prior activation. Since survivin suppresses NK cells activity,^103^ its reduction by the peptide likely underlies this increased infiltration (Figures 7A and 7B).

It should be noted that although nude mice are immunocompromised, they can support extrathymic T cell development. Several sites—including the liver, intestine, salivary glands, uterus, lymph nodes, and spleen—enable T cell maturation in both mice and humans.^120^^,^^121^ The liver, for instance, serves as a niche for T cell development in athymic mice,^122^ while human tonsils support a maturation pathway from CD34^+^CD38ˆdimˆLin^−^ progenitors to CD3^+^ T cells.^123^ Functional TCRαβ^+^ T cells have also been reported to develop extrathymically in the spleen and lymph nodes of bone marrow transplant recipients.^124^

Tumors often become immunosuppressive via mechanisms like hypoxia,^125^ stemness,^126^ and mesenchymal cell presentation.^127^ This escape can be countered by immune checkpoint inhibitors (ICIs) such as antibodies targeting CTLA-4, and PD-1 or PD-L1. Unfortunately, only about 15%–25% of cancer patients including NSCLC,^128^ respond to such treatments^129^^,^^130^ and can develop resistance to treatment.

PD-1, typically expressed by immune cells, is also found in many cancers, including NSCLC.^131^^,^^132^ As found previously with the nucleus targeted survivin/BIRC5-derived peptide,^66^ we showed that peptides targeted to the mitochondria also increased PD-1 and PD-L1 expression in tumor cells, with the highest increase seen with the nucleus-targeted peptide (Figures 7C–7G). While the regulatory mechanism by which the peptide induces their expression remains to be clarified, it may involve multiple signaling pathways such as NF-κB, MAPK, mTOR, STAT, and c-myc.^133^^,^^134^ Survivin has been reported to interact with STAT-3^52^^,^^135^ and Myc.^136^^,^^137^

In this context, DNA-damaging agents like ionizing radiation and cisplatin upregulate PD-L1 by impairing DNA repair.^138^^,^^139^ Similarly, the peptide may promote PD-L1 expression by reducing nucleus survivin, compromising DNA repair.

The peptide also increased PD-1 expression on both tumor cancer cells and infiltrating immune cells (Figures 7E–7G). Interestingly, as in our peptide, cisplatin significantly increased PD-1 expression in chemo-surviving NSCLC cells.^140^

Moreover, recent studies showed that PD-1 also expressed an intrinsic variant (iPD-1) in cancer cells, where it plays important roles in tumor progression. iPD-1 is widely expressed in tumor tissues and cancer cell lines.^91^ It is found in various cancers, including melanoma, HCC, and NSCLC, and may function in non-immune cells, suggesting that the protein may also function in non-immune cells.^90^^,^^141^^,^^142^ Its role varies by cancer type—suppressing tumorigenesis in NSCLC and colon cancer, but promoting it in melanoma, glioblastoma, and hepatocellular carcinoma (HCC).

In NSCLC, PD-1 overexpression decreased cancer cell viability, while PD-1 depletion promoted cell growth and resistance to anti-PD-1/PD-L1 therapies.^90^^,^^141^ This immune activation suggests that the survivin-derived 1H13-Nuc peptide may enhance anti-tumor immunity and sensitize tumors to immune checkpoint inhibitors, potentially converting immune-cold tumors into responsive ones.

### Survivin as a target for cancer therapy using a survivin-derived peptide

Survivin, due to its multifunctionality and overexpression in most cancers but not in adult non-proliferating tissues, makes it a promising therapeutic target.^94^^,^^112^^,^^143^ Our survivin/BIRC-derived peptide, mimics survivin’s dimerization, tubulin, and CPD binding sites, disrupts its multiple functions and reduces its expression, thereby affecting survivin-associated protein activities (Figure 8).

By targeting survivin in the cytosol, nucleus, and mitochondria, the peptide inhibits its anti-apoptotic role, involving disruption of its protein interactions and decreases the expression of survivin (Figure 8A). Survivin in the nucleus^3^^,^^16^^,^^111^ regulates chromosome alignment and segregation during mitosis and cytokinesis,^112^ with the peptide interfering with these survivin functions, arresting cell-cycle progression and proliferation (Figure 8B). The peptide also enhances tumor infiltration of CD8^+^ and NK cells and increases PD-1 and PD-L1 levels in cancer cells, boosting anti-tumor immunity (Figure 8C).

Despite decades of research, survivin-targeting therapies have struggled due to poor specificity, toxicity, and delivery issues.^43^^,^^57^^,^^58^^,^^59^^,^^60^^,^^61^^,^^62^^,^^63^^,^^64^^,^^65^^,^^144^^,^^145^^,^^146^^,^^147^ Thus, there is an urgent need to develop new anti-survivin strategies that are more practical for therapeutic use in human patients.

Various strategies have been explored to target survivin, including inhibitors of its interactions with partner proteins, homodimerization, gene transcription, mRNA, immunotherapy, and proteasomal degradation.^43^^,^^57^^,^^58^^,^^59^^,^^60^^,^^61^^,^^62^^,^^63^^,^^64^^,^^65^^,^^145^^,^^146^^,^^147^^,^^148^ Small molecules like YM155 showed off-target effects, while nucleic acid-based therapies and vaccines faced challenges with potency, immune evasion, or patient variability.^43^^,^^65^^,^^146^^,^^147^ FL118, an analog of a bioactive alkaloid camptothecin, downregulates survivin, but also affects several anti-apoptotic proteins (Mcl-1, XIAP, and cIAP2) and inhibits topoisomerase-1^149^. These off-target effects lead to unpredictable toxicity and limited therapeutic indices for small-molecule approaches.

In addition, nucleic acid-based approaches to suppress BIRC5 mRNA have struggled with delivery and limited efficacy.^43^^,^^62^^,^^145^ LY2181308, an antisense oligonucleotide, reduced tumor survivin by only ∼20% in clinical trials.^150^ Immunotherapies, like the SurVaxM vaccine, showed some benefit, but are hindered by immune evasion, HLA restrictions, and the need for strong patient immune responses, which are often lacking in advanced cancer.

In another approach, *in silico* screening identified a small molecule, LQZ-7F, which induced proteasome-dependent survivin degradation, mitotic arrest, and apoptosis; disrupted microtubule structures; and blocked human tumor growth in mouse xenograft models.^44^^,^^151^ However, clinical translation has been limited by off-target effects and toxicity. These setbacks highlight the difficulty of effectively targeting survivin with conventional approaches.

In contrast, our novel cell-penetrating peptide is the first compound derived from survivin. It mimics the survivin dimerization interface and acts as a “decoy” peptide to disrupt its dimerization and interactions with partners localized in the mitochondria, cytosol, and nucleus, such as SMAC and tubulin. Unlike previous strategies, it bypasses the need for mRNA delivery or immune presentation and offers high specificity with reduced toxicity, providing a practical and effective new approach to survivin-targeted cancer therapy.

In conclusion, the 1H13-Nuc peptide, derived from the native survivin sequence with minimal modifications, shows high specificity and low off-target effects. By disrupting survivin dimerization and its interactions with partners, and reducing survivin expression, it induces apoptosis and effectively impairs multiple stages of cell division. Thus, this peptide offers a promising new approach to target survivin, addressing long-standing challenges that have limited previous therapeutic efforts.

## Materials and Methods

Materials, sulforhodamine B (SRB) cell proliferation assay, protein extraction from tumors, immunoblotting, and RNA preparation, RT-qPCR analysis are all described in the Supplemental Materials.

### Peptides

Peptides were synthesized by GL Biochem (Shanghai, China) to >95% purity. They were first dissolved in DMSO as a 20 mM solution and then diluted 20-fold in water or the appropriate buffer.

### Purified survivin, SMAC, and tubulin proteins

Purified human recombinant SMAC (10339-H08E) and human survivin (10356-HNCE) were produced by Sino Biologicals (Wayne, PA). Purified porcine tubulin was obtained from Cytoskeleton Inc. NeutrAvidin was obtained from (Life Technologies).

### Cell culture and peptide treatment

A549 (human lung adenocarcinoma epithelial), MDA-MB-231 (human breast cancer), PC-3 (human prostate adenocarcinoma epithelial), U-87MG (human glioblastoma), SH-SY5Y (human neuroblastoma), HeLa (human cervix adenocarcinoma), Jurkat (human acute T cell leukemia), K562 (human chronic myelogenous leukemia), and KMH2-LC (human anaplastic thyroid carcinoma) cell lines were obtained from the American type culture collection (ATCC, Manassas, VA) and maintained according to ATCC instructions. A549, MDA-MB-231, PC-3, U-87MG and SHSY5Y cell lines were cultured in DMEM supplemented with 10% fetal bovine serum (FBS), 100 U/mL penicillin, and 100 μg/mL streptomycin. Jurkat, K562, and KMH2-LC cells were cultured in RPMI-1640 medium.

HUV-EC-C (human vascular endothelial cells) were obtained from the Japanese Collection of Research Bioresources (JCRB) Cell Bank (Osaka, Japan) and cultured in DMEM/F-12 (HAM) medium (Sartorius, Göttingen, Germany) supplemented with 10% FBS.

The cells were maintained in a humid atmosphere at 37°C and 5% CO_2_ and were routinely tested for mycoplasma contamination.

For the treatment with peptides, cells at approximately 80% confluence were incubated in serum-free medium with various concentrations of the peptide of interest for 24 h at 37°C in the presence of 5% CO_2_. The cells were then centrifuged (1500 x g, 5 min), washed twice with PBS, and subjected to the intended assays.

### Peptide array and SMAC binding to glass-bound peptides derived from SMAC-interacting proteins

Customized 768-peptide sequences derived from 11 SMAC-interacting proteins were arrayed on a glass slide by INTAVIS peptide services (GmbH & Co. KG; Tübingen, Germany). It comprised 768 peptide sequences derived from 11 SMAC-interacting proteins including HTRA2 (serine peptidase 2), TRAF-2 (TNF receptor associated factor 2), MTFR1 (mitochondrial fission regulator 1), BIRC2 (cIAP1, baculoviral IAP repeat-containing protein 2), and BIRC5/Survivin (baculoviral IAP repeat-containing protein 5). Each peptide was composed of 25 amino acids in length, with an overlap of 15 amino acids with the peptides before and following it. To detect SMAC interacting peptides, the peptide array was incubated with purified SMAC and then with anti-SMAC antibodies, followed by HRP-conjugated secondary antibody that was detected by the reaction product’s chemiluminescence. Briefly, after washing (3 times, 10 min each) with tris-buffered saline (150 mM NaCl, 50mM tris-HCl, pH 7.4), the array slides were incubated overnight with blocking buffer (tris-buffered saline containing low-fat dry milk, 2.5%, w/v). The slides were then incubated for 4h or overnight with purified SMAC (0.8 μM) in blocking buffer at room temperature (RT). Following extensive washing with tris-buffered saline containing 0.05% Tween 20, SMAC binding was detected by using anti-SMAC antibodies with HRP-conjugated anti-rabbit IgG as a secondary antibody (Table S1). The blots were developed using a chemiluminescent substrate (Advantsa; San Jose, CA) according to the manufacturer’s instructions.

### Microscale thermophoresis (MST) assay

MST analysis was performed using a NanoTemper Monolith NT.115 apparatus, as previously described.^152^ Briefly, purified survivin was fluorescently labeled using a NanoTemper Protein Labeling Kit BLUE-NHS (L001, NanoTemper Technologies). A constant concentration of survivin (650 nM) was incubated (30 min at 37°C) with several concentrations of purified survivin, tubulin, SMAC, or 1H13 peptide in 10 mM Tricine, pH 7.4, and 20 mM NaCl buffer. Then 3–5 μL of the samples were loaded into MST-grade glass capillaries, and thermophoresis was measured using a Monolith-NT115 apparatus, and the thermophoresis was analyzed (LED 20%, 40%, and 80%; IR laser 80%).

### Assessment of apoptosis and cell death

Cell death was analyzed by propidium iodide (PI) staining (final concentration of 6.25 μg/mL), followed by flow cytometry with an iCyt sy3200 Benchtop Cell Sorter/Analyzer (Sony Biotechnology Inc.; San Jose, CA) and analysis with EC800 software. Apoptosis was analyzed by PI and Annexin V-FITC staining, carried out according to the manufacturer’s instructions. After treatment, cells were harvested (1,500 g, 5 min), washed, and re-suspended in 200 μL of binding buffer (10 mM HEPES/NaOH, pH 7.4, 140 mM NaCl, and 2.5 mM CaCl_2_). Annexin V-FITC/PI staining was performed, and the samples were analyzed by flow cytometry. At least 10,000 events were recorded, represented as dot plots.

### Fluorescein isothiocyanate (FITC)-labeled peptides and cell subcellular localization

Peptide-FITC labeling was carried out by peptide 1H13-Nuc (1mg) incubation with 50 μM FITC (Sigma; St. Louis, MO) for 30 min in 10 mM Tricine, pH 8.7, at 37°C. Unreacted reagent was removed by dialysis using membranes with a cut-off of 2,000 Da (Vivaspin 2 Membrane, Sartorius; Göttingen, Germany).

For FITC-peptide cell sub-cellular localization, A549 cells (30,000 cells/well) were seeded on sterile glass coverslips placed on 12-well cell culture plates, and after 24 h, the cells were incubated for 90 min in a serum-free medium with 20 μM FITC-labeled peptide. They were then washed with PBS and fixed with 4% paraformaldehyde (15 min, RT). Cells were incubated with blocking buffer for 2 h, then with anti-SMAC antibodies (mitochondria), anti-IP3R (ER) or with anti-GM130 (golgi) overnight at 4°C. The cells were then washed and incubated in the dark with secondary antibodies (2 h, RT), stained with DAPI (nucleus), washed, mounted with fluoroshield mounting medium (Immunobioscience; Mukilteo, WA), and imaged by confocal microscopy (Olympus 1X81).

### Crosslinking experiments

The conditions for crosslinking were selected based on previous study^153^ Cells were grown in 6-well plates (60% confluence), subjected to peptide treatment as indicated in the figure legends, harvested, and washed with PBS, and their protein concentration was determined. Cells (1 mg of protein/ml) were incubated at 30^o^C with the crosslinking reagent EGS (100 μM) in PBS at pH 8.3 for 15 min.

Crosslinking was also performed with purified survivin protein (33 μg/mL) and in the absence and the presence of 1H13 peptide (30 or 60 μM) in PBS, pH 8.3. Following incubation (15 min, 30^o^C), samples were subjected to crosslinking with EGS (50 or 100 μM, 15 min, 30^o^C). 1H13 peptide (30 μM and 60 μM).

The cells (20–30 μg protein) and survivin (1 μg) samples were then subjected to SDS-PAGE (4–20% acrylamide) and immunoblotted with survivin-specific antibodies to visualize their oligomerization. Nitrocellulose membranes were treated with 0.1 mM glycine at pH 2.2 prior to immunoblotting and washed several times with 0.1% Tween 20 in tris-buffered saline (TBST). Enhanced chemiluminescent substrate (Advantsa; San Jose, CA) was used to detect HRP activity. Peptide samples were subjected to SDS-PAGE (4%–20% acrylamide) and Coomassie blue staining. Quantitative analysis of immunoreactive survivin bands or peptide bands was performed using ImageJ software (Bethesda, MD).

### Mouse xenograft model

Female athymic nude mice (6–8 weeks old) were procured from Envigo (Indianapolis, IN). Mice were housed (4 animals per cage) with a 12/12 h light/dark cycle and with *ad libitum* access to food and water.

All experiments were carried out in agreement with the ethical committee guidelines following the approval of the Ben-Gurion University and Israel state Institutional Animal Care and Use Committees.

For the human lung cancer A549 xenograft model, cells (7x10^6^ cells/mouse) were implanted subcutaneously on the dorsal flanks. Tumor growth was recorded using digital calipers, and tumor volume was calculated as follows: X2 × Y/2, where X and Y are the short and long tumor dimensions, respectively.

Mice were divided randomly into five groups once the average tumor volume reached ∼60–100 mm^3^. The experimental groups included: Group 1 (vehicle control), Group 2 (1H13-Mito), Group 3 (2/3D-1H13-Mito), Group 4 (2/3D-1H13-Antp), and Group 5 (2/3D-1H13-Nuc). All animals were treated twice a week intravenously (final peptide concentration of 20 mg/kg).

For the human triple negative breast cancer MDA-MB-231 xenograft model, cells (3x10^6^ cells/mouse) were implanted subcutaneously on dorsal flanks. Tumor formation was followed, and when reaching a volume of 50–100 mm^3^ (day 14), the mice were split into two tumor volume-matched groups and were intravenously injected every 4 days with the vehicle (NaCl 0.9%) or with paclitaxel (10 mg/kg), and tumor volume was followed.

In both experiments, mice were sacrificed at the end of the experiment, and the tumors were dissected and weighed. Then tumors were fixed and processed for immunofluorescence.

### TUNEL assay

Paraffin-embedded tumor sections were processed for a TUNEL assay using the DeadEnd Fluorometric TUNEL assay (Promega; Madison, WI) according to the manufacturer’s instructions. Sections were deparaffinized, equilibrated in PBS, permeabilized with proteinase K (20 μg/mL in PBS), post-fixed in 4% paraformaldehyde, and incubated in TdT reaction mix for 1 h at 37°C in the dark. The slides were then washed in saline-sodium citrate buffer and counter-stained with propidium iodide (1 μg/mL). After mounting the slide with Fluoroshield mounting medium (Immunobioscience; Mukilteo, WA), images were collected using a confocal microscope (Olympus IX81).

### Immunofluorescence (IF)

Cells were seeded on sterile glass coverslips in 12-well plates and cultured until reaching around 80% confluence. Cells were treated with the desired peptide as indicated in the figure legends, in a serum-free,^66^ growth media for 24 h. Then cells were washed with PBS, fixed with 4% paraformaldehyde (20 min), washed three times with PBS, permeabilized with 0.3% Triton X-100 in PBS (PBST), and blocked with blocking buffer (1% fatty acid-free BSA diluted in PBS) for 2 h. Cells were probed with the desired primary antibodies (Table S1) in an antibody solution containing 1% fatty acid-free BSA in PBST and incubated overnight at 4°C. The next day, cells were washed three times with PBS and probed with fluorescent-conjugated secondary antibody for 2 h at RT in the dark. Following a wash with PBS, coverslips were incubated with DAPI (0.5 μg/ml) for 15 min in the dark, and carefully washed, dried, and mounted on slides with Fluoroshield mounting medium (Immunobioscience, Mukilteo, WA, USA).

Formalin-fixed and paraffin-embedded 5-μm-thick tumor tissue sections were deparaffinized by heating the slides on a 60°C hot-plate for 1 h and using xylene. Thereafter, the sections were rehydrated using a graded ethanol series (100%–50%) and subjected to antigen retrieval using either 0.01 M citrate buffer (pH 6.0) or 0.01 M tris-EDTA (pH 9) at 95°C–98°C for 30 min. The sections were incubated in blocking buffer (10% NGS, 1% BSA, and 0.1% Triton) for 2 h, and then incubated with primary antibodies in an antibody buffer (5% NGS, 1% BSA) (Table S1) overnight at 4°C. After washing with PBS containing 0.1% Triton (PBST), sections were incubated with fluorescent-tagged secondary antibodies (Table S1) for 2 h at room temperature in the dark. Following a wash with PBST, sections were incubated with DAPI for 15 min in the dark, washed, mounted with Fluoroshield mounting medium.

All images were acquired using a confocal microscope (Olympus 1X81) under identical exposure settings to ensure consistency across samples. Fluorescence intensity measurements were performed under the same acquisition parameters, and quantification was conducted using a comparable number of cells per condition. This approach ensured that differences in fluorescence signals reflect biological variation in protein levels. Image analysis was performed using standardized software and protocols. ImageJ (Bethesda, MD) software was used to quantify the signal intensity in the images, represented as the relative level per cell.

## Statistics

Results are presented as the means ± SEM of results obtained from three or more independent experiments. A difference was considered statistically significant when the *p* value was <0.05 (∗), <0.01 (∗∗), <0.001 (∗∗∗), or <0.0001 (∗∗∗∗), as assessed by an unpaired Student’s two-tailed *t* test.

## Data and code availability

All data are available in the main text or the Supplementary Materials.

## Acknowledgments

This research was funded by the National Institute of Biotechnology in the Negev (NIBN), Ben Gurion University and the Krieger Foundation.

## Author contributions

M.S., V.B, A.S.-K., and S.K.P., carried out experiments, data analysis, and figure preparation. G.R. reviewed the results and the M.S. and L.G. supervised part of the data analysis and presentation, and M.S. and V.S.-B. supervised the study provided experimental oversight, interpreted the results, arranged the figure presentations, and wrote the manuscript.

## Declaration of interests

The corresponding author has a connection with RAD Therapeutics.

## Supplemental information

Document S1. Figures S1–S7 and Tables S1–S3

Document S2. Article plus supplemental information
