# Survivin/BIRC5-derived peptide disrupts survivin dimerization and cell division and induces multifaceted anti-cancer effects

**DOI:** 10.1016/j.omton.2026.201142

**Published:** 2026-01-30

**Authors:** Manikandan Santhanam, Venkatadri Babu, Anna Shteinfer-Kuzmine, Swaroop Kumar Pandey, Larisa Gheber, Gilead Raday, Varda Shoshan-Barmatz

## Main text

(Molecular Therapy: Oncology *34*, 1−25; March 2026)

In the originally published version of this article, KMH2-LC cells were incorrectly described as human anaplastic thyroid carcinoma but should be KMH2 (human Hodgkin lymphoma). The authors apologize for this error.

