# Supplementary material for: Survivin/BIRC5-derived peptide disrupts survivin dimerization and cell division and induces multifaceted anti-cancer effects
Source: Mol Ther Oncol. 2026 Jan 3;34(1):201123. doi: 10.1016/j.omton.2025.201123 (PMC12857549; doi:10.1016/j.omton.2025.201123)
Supplement: Document S1. Figures S1–S7 and Tables S1–S3 [file mmc1.pdf]

OMTON, Volume *34*

Supplemental information

Survivin/BIRC5-derived peptide disrupts survivin dimerization and cell division and induces multifaceted anti-cancer effects

Manikandan Santhanam, Venkatadri Babu, Anna Shteinfer-Kuzmine, Swaroop Kumar Pandey, Larisa Gheber, Gilead Raday, and Varda Shoshan-Barmatz

Supplementary Materials

Survivin/BIRC5-derived peptide disrupts survivin dimerization and cell division and induces multifaceted anti-cancer effects

Manikandan Santhanam[1], Venkatadri Babu[1], Anna Shteinfer-Kuzmine[2], Swaroop Kumar Pandey[1*], Larisa Gheber[3], Gilead Raday[4], and Varda Shoshan-Barmatz[1,2#]

[1]Department of Life Sciences; [2]The National Institute for Biotechnology in the Negev; [3]Department of Chemistry, Ben-Gurion University of the Negev, Beer Sheva, Israel; [4]RAD Therapeutics Inc. USA

.

# Corresponding author:

Varda Shoshan-Barmatz

Department of Life Sciences

Ben-Gurion University of the Negev and the National Institute for Biotechnology in the Negev

Beer Sheva, Israel

vardasb@bgu.ac.il

* Current address: Department of Biotechnology GLA University, Mathura, India 281406

Materials and Methods

Materials

Propidium iodide (PI), sulforhodamine B, bovine serum albumin (BSA), trypan blue, 4',6-diamidino-2-phenylindole (DAPI), fluorescein isothiocyanate (FITC), Triton X-100, and Tween-20 were obtained from Sigma (St. Louis, MO). Paraformaldehyde and formaldehyde were from Emsdiasum (Hatfield, PA). Phosphate-buffer saline (PBS), Dulbecco's modified Eagle's medium (DMEM), normal goat serum (NGS), the supplement fetal bovine serum (FBS), and penicillin-streptomycin were purchased from Gibco (Grand Island, NY). EGS was obtained from Pierce (Appleton, WI). Annexin V-fluorescein isothiocyanate (FITC) was from Enzo Life Sciences (Lausanne, Switzerland). Protease inhibitor cocktail set III was obtained from Calbiochem (Nottingham, UK). A TUNEL assay kit was from Promega (Madison, WI). Dimethyl sulfoxide (DMSO) was purchased from MP Biomedicals (Solon, OH). Paclitaxel was provided by Soroka Medical Center (Beer-Sheva, Israel). Fluoroshield was obtained from Immuno Bio Science Corporation (Washington, DC). Horseradish peroxidase (HRP)-conjugated secondary antibodies and primary antibodies were obtained from different sources; sources and dilutions are detailed in Table S1.

Sulforhodamine B (SRB) cell proliferation assay

Cells were seeded in 96-well cell culture plates (8,000/well) and allowed to grow for 24h. They were incubated without or with the indicated concentration of the peptide for 24h in serum-free medium. After washing with PBS, the cells were fixed with 10% trichloroacetic acid (TCA) for 1h, and stained with 0.4% SRB for 20–30 min. Excess SRB was removed, and the cells were washed with 1% acetic acid. SRB extraction was done using 100 mM tris-base, and absorbance at 510 nm was determined using an Infinite M1000 plate reader (Tecan; Männedorf, Switzerland).
used to quantify the signal intensity in the images, represented as the relative level per cell.

Protein extraction, gel electrophoresis, and immunoblotting

Cells subjected to peptide treatment were lysed using lysis buffer (50 mM Tris-HCl, pH 7.5, 150 mM NaCl, 1 mM EDTA, 1.5 mM $MgCl_2$, 10% glycerol, 1% Triton X-100), supplemented with a protease inhibitor cocktail (Calbiochem; San Diego, CA). The lysates were vortexed and incubated for 15 min on ice. After incubation, they were centrifuged at 15,000 g for 10 min at 4°C, and the protein concentration of the supernatant was measured. Protein samples were stored at -80°C until further use in gel electrophoresis. Protein aliquots (10–20 μg) were subjected to SDS-PAGE and then were electro-transferred onto nitrocellulose membranes for immunostaining. The membranes were first blocked by incubation (2h) with a solution containing 5% non-fat dry milk and 0.1% Tween-20 in tris-buffered saline (TBST, pH 7.8), followed by incubation with primary antibodies (as listed in Table 1). The membranes were then incubated with HRP-conjugated anti-mouse or anti-rabbit IgG as secondary antibodies. Enhanced chemiluminescent substrate (Advantsa; San Jose, CA) was used to visualize HRP activity. Band intensities were analyzed using ImageJ (Bethesda, MD) software.

RNA preparation, q-RT-PCR analysis

Total RNA was isolated from A549 cells treated with or without the peptide using Trizol reagent (Sigma; St. Louis, MO). The RNA was reverse transcribed into complementary DNA (cDNA) with the

PCRBio cDNA synthesis kit (PCR Biosystems, Wayne, PA, USA) and used for real-time q-RT-PCR using commercially synthesized specific primers (Table S2) with Power SYBER green master mix (Applied Biosystems, Foster City, CA), according to the manufacturer's instructions.

The levels of target genes were normalized relative to b-actin mRNA levels. Samples were amplified by a 7300 Real Time PCR System (Applied Biosystems) for 40 cycles using the following PCR parameters: 95°C for 15 s, 60°C for 1 min, and 72°C for 1 min. Relative expression levels for each gene in each sample were calculated by the ddCT-based calibrated standard curve method. The results represent the mean values ± SEM (n=3).

Table S1. Antibodies used in the study

Antibodies against the specific protein, source, catalogue number, and dilutions used in immunofluorescence (IF) and immunoblot (WB) are presented.

Antibody	Source and Catalog. No.	Dilution	
		IF	WB
Mouse monoclonal anti-SMAC/Diablo	Cell Signaling Technology, Inc; Boston, MA, 2954s	1:400	-
Rabbit polyclonal anti-SMAC/Diablo	Abcam; Cambridge, UK, ab8115	1:500	1:1,000
Rabbit monoclonal anti-survivin antibody	Abcam; Cambridge, UK, ab134170	1:800	1:1,000
Mouse monoclonal anti-survivin	Santa Cruz Biotechnology; Dallas, TX, sc-17779	1:200	1:500
Rabbit polyclonal anti-P53	Abcam; Cambridge, UK, ab131442	1:200	1:1,500
Rat monoclonal anti-mouse CD-8	Biolegend; San Diego, CA, 100732	1:50	-
Rabbit monoclonal anti-β-tubulin	Abcam; Cambridge, UK, ab179513	1:1000	
Rat monoclonal PE anti-mouse CD49b (pan-NK cells)	Biolegend; San Diego, CA DX5	1:100	-
Purified Mouse Anti-GM130	BD Transduction Laboratories, Cat. 610822	1:200	-
Rabbit polyclonal anti-ATP Synthase 5A	Abcam, Cambridge, UK, ab151229	1:200	-
Rabbit anti-IP3 receptor	Abcam, Cambridge, UK, ab5804	1:200	-
Human monoclonal anti-PD-L1	Merck Sharp & Dohme Corp.; Rahway, NJ, Keytruda	1:100	-
Mouse monoclonal to PD-1	Abcam; Cambridge, UK, ab52587	1:200	-
Rabbit monoclonal anti-PD-1	Abcam; Cambridge, UK, ab214421	1:100	-
Rabbit monoclonal anti-Ki-67	Abcam; Cambridge, UK, ab16667	1:1,000	-
Anti-mouse IgG, Alexa Fluor 488	Abcam; Cambridge, UK, ab150113	1:1,000	-
Anti-mouse IgG, Alexa Fluor 555	Abcam; Cambridge, UK, ab150114	1:1,000	
Anti-rabbit IgG, Alexa Fluor 555	Abcam; Cambridge, UK, ab150078	1:1,000	-
Anti-rabbit IgG, Alexa Fluor 488	Abcam; Cambridge, UK, ab150077	1:1,000	-
Anti-Human IgG, Alexa Fluor 594	Jackson Immuno Research; West Grove, PA, 609-585-213	1:1,000	-
Anti-rat IgG, Alexa Fluor 488	Abcam; Cambridge, UK, ab150153	1:1,000	-

Table S2. Real-time PCR primers used in this study
The genes examined and the forward and reverse sequences of the primers used are indicated.

Gene	Primer sequences
β-Actin (human)	Forward: 5' ACTCTTCCAGCCTTCCTTCC 3'<br>Reverse: 5' TGTTGGCGTACAGGTCTTTG 3'
KI-67(human)	Forward: 5' GAAAGAGTGGCAACCTGCCTTC 3'<br>Reverse: 5' GCACCAAGTTTTACTACATCTGCC 3'
P53 (human)	Forward: 5' CCTCAGCATCTTATCCGAGTAGTGG 3'<br>Reverse: 5' TGGATGGTGGTACAGTCAGAGC 3'

Table S3. Survivin-interacting proteins, their function, and cellular localization
Proteins proposed to interact with survivin, their function, and cellular localization are indicated.

No	Protein	Function (location)	Ref.
1	**Chromosomal passenger complex (CPC)**	With survivin, borealin, the aurora-B kinase, and INCENP form a complex essential for correct chromosome alignment, centromere localization, spindle assembly checkpoint signaling and cytokinesis, and mitotic function of the CPC.	[1]
2	**Aurora kinases**	Survivin forms a complex with Aurora kinases that regulates mitosis and helps in proper chromosome alignment and segregation during cell division.	[2-4]
3	**Borealin (CDCA8)**	Borealin and Survivin form a subcomplex that helps recruit the entire CPC to centromeres during early mitosis. The interaction also stabilizes binding of CPC to chromatin and helps facilitate its dynamic relocation throughout mitosis. Borealin acts as a scaffold, allowing INCENP to bind and bring in the aurora-B kinase.	[1]
4	**INCENP**	The N-terminal BIR domain of survivin interacts with the C-terminal regions of INCENP, which helps localize and activate aurora-B kinases.<br>Survivin and INCENP co-localize to the centromeres and spindle midzone during mitosis. **(cytoplasm, nucleus)**	[1]
5	**CASP3, CASP7, CASP9**	These caspases are involved in the activation cascade of caspases responsible for apoptosis execution. Proximity ligation assay and *in-silico* (protein–protein docking and molecular dynamics simulation) are methods used to understand survivin interactions with CASP-3, CASP-7, and CASP-9. It was found that survivin interacts with the catalytic site and/or at the dimerization site of CASP-3 and CASP-7 **(cytoplasm)**	[5]
6	**Human IAP family member cIAP2, X chromosome-linked IAP (XIAP, birc4), apollon/bruce (birc6)**	Inhibitor of the apoptosis (IAP) protein family, acting as a direct caspase inhibitor. Regulates caspases and apoptosis, modulates inflammatory signaling and immunity, mitogenic kinase signaling, and cell proliferation, as well as cell invasion and metastasis. Complexes between survivin and XIAP including c-IAP1 and BRUCE have been reported. IAP complexes may provide a general mechanism to expand the functional repertoire of these molecules. **(cytoplasm, nucleus, plasma membrane**)	[6, 7]
7	**Beclin 1**	Beclin 1, a key regulator of autophagy that regulates the formation of autophagosome. Beclin interacts with survivin and regulates the sensitivity of human glioma cells to TRAIL-induced apoptosis. Knockdown of Beclin 1 resulted in down-regulation of survivin protein. **(cytoplasm)**	[8]
8	**BCL2L1 (BCL2 like 1)**	BCL2L1, a Potent inhibitor of cell death. Inhibits activation of caspases. Also acts as a regulator of the G2 checkpoint and progression to cytokinesis during mitosis. Evidence for direct physical interaction is limited. Some studies suggest that survivin can co-immunoprecipitate with Bcl-2, indicating possible complex formation under certain conditions. **(cytoplasm, nucleus, mitochondria)**	[9]

9	**STAT3 (Signal transducer and activator of transcription 3)**	Signal transducer and transcription activator that mediates cellular responses to interleukins and other growth factors. Plays an apoptotic role by transctivating survivin expression under LEP activation. Using proteomic and co-immunoprecipitation approaches, it was demonstrated that survivin and STAT3 physically interact. **(cytoplasm, nucleus)**	[10]
10	**SRC Proto-oncogene tyrosine-protein kinase sarcome**	Non-receptor protein tyrosine kinase. Participates in signaling pathways that control a diverse spectrum of biological activities including gene transcription, immune response, cell adhesion, cell-cycle progression, apoptosis, migration, and transformation. SRC can phosphorylate survivin on specific tyrosine residues, affecting its stability and anti-apoptotic function. Phosphorylated survivin has an enhanced ability to inhibit apoptosis and promote tumor cell survival. Survivin can co-immunoprecipitate with SRC in certain cancer cell lines, indicating a physical interaction or association in protein complexes. **(mitochondria, cytoskeleton, plasma membrane, nucleus)**	[11]
11	**MYC**	Proto-oncogene and transcription factor that activates the transcription of growth-related genes. Survivin forms a complex with c-Myc, as demonstrated by co-immunoprecipitation. MYC directly upregulates survivin expression by binding to its promoter. Survivin protects MYC from degradation. Overexpression of survivin enhances MYC-driven oncogenicity, while survivin knockdown reduces MYC-induced proliferation and tumor formation**, (nucleus)**	[12] [13]
12	**CDK1, CDK4 (*Cyclin-dependent kinase 1, 4)***	Plays a key role in the control of the eukaryotic cell cycle by modulating the centrosome cycle, and mitotic onset. Promotes G2-M transition and regulates G1 progress and G1-S transition via association with multiple interphase cyclins. Survivin interacts with various CDKs, particularly CDK1 and CDK4, which are involved in cell-cycle regulation. Nuclear translocation of survivin is followed by an interaction with CDK4. Survivin competitively binds with the CDK4/p16INK4a complex to initiate S-phase progression from the G1 phase. These interactions influence cell-cycle progression and mitosis. CDK1 phosphorylates survivin during mitosis, particularly at threonine 34. This phosphorylation is essential for survivin's anti-apoptotic function and its role in mitotic progression. Phosphorylation by CDK1 stabilizes survivin and promotes its interaction with other components of the CPC such as the aurora-B kinase. Survivin initiates cell cycle entry through its competitive interaction with Cdk4 and Cdk2/cyclin E complex activation. **(mitochondria, cytoplasm, nucleus)**	[14-17]
13	**SMAC/Diablo (Second mitochondria-derived activator of caspase)/direct IAP-binding protein with low pI)**	Overexpressed in several types of cancers including lung cancer. It is a protein that antagonizes IAPs, including survivin/BIRC5, which binds to SMAC/Diablo and prevents caspase activation, thereby leading to negative regulation of apoptosis. Inhibition of this interaction promotes apoptosis. SMAC depletion leads to reduced tumorogenesis in lung cancer xenografts via modulation of phosphatidylethanolamine synthesis by the PSD enzyme. SMAC/Diablo also interacts with survivin. **(mitochondria, cytoplasm, nucleus)**	[18-25]
14	**PSD (Phosphatidyl-serine decarboxylase)**	Catalyzes the formation of phosphatidylethanolamine from phosphatidylserine. Interacts with survivin and inhibits its activity. **(mitochondria)**	[22]
15	**Non-muscle myosin II (NMII)**	Survivin binds to non-muscle myosin II (NMII), regulating its filament assembly. Direct interaction between survivin and myosin II is required for cytokinesis. (**cytoplasm)**	[26]
16	**Tubulin**	Survivin interacts with tubulin during mitosis, localizes to the mitotic spindle and, thus, regulates mitosis. (**cytoplasm, nucleus)**	[27, 28]

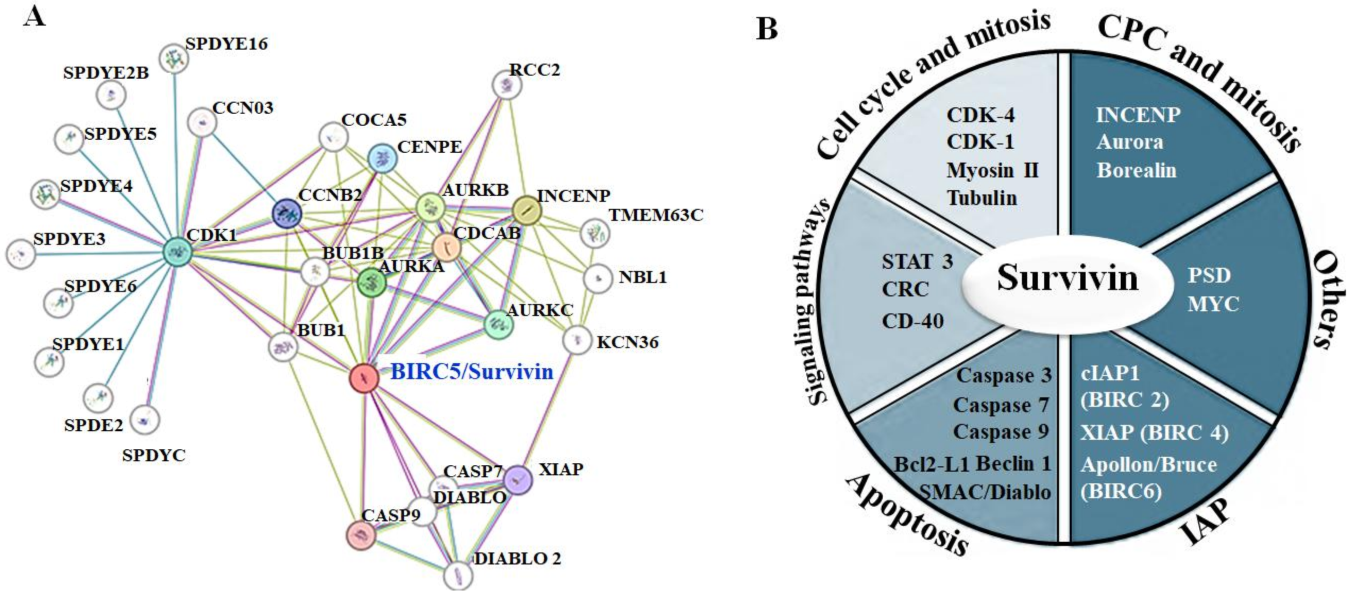

Fig. S1. Human proteins interacting with surviving

(A) A STRING network analysis reveals a wide array of interactions involving survivin. The interaction map was generated using the STRING database, and includes both direct and indirect associations—such as proteins that regulate survivin expression, share signaling pathways, or are functionally linked without direct physical binding. Interaction evidence is categorized using seven color-coded lines: red (gene fusion), green (genomic neighborhood), blue (gene co-occurrence), purple (experimental data), yellow (text mining), light blue (curated databases), and black (co-expression). (B) Proteins shown to directly interact with survivin, grouped by function. These physical interactions were validated by various experimental techniques including proximity-dependent biotin identification (BioID), proximity ligation assay (PLA), anti-tag co-immunoprecipitation, protein–protein docking, molecular dynamics simulation, and X-ray crystallography, as summarized in Table S3.

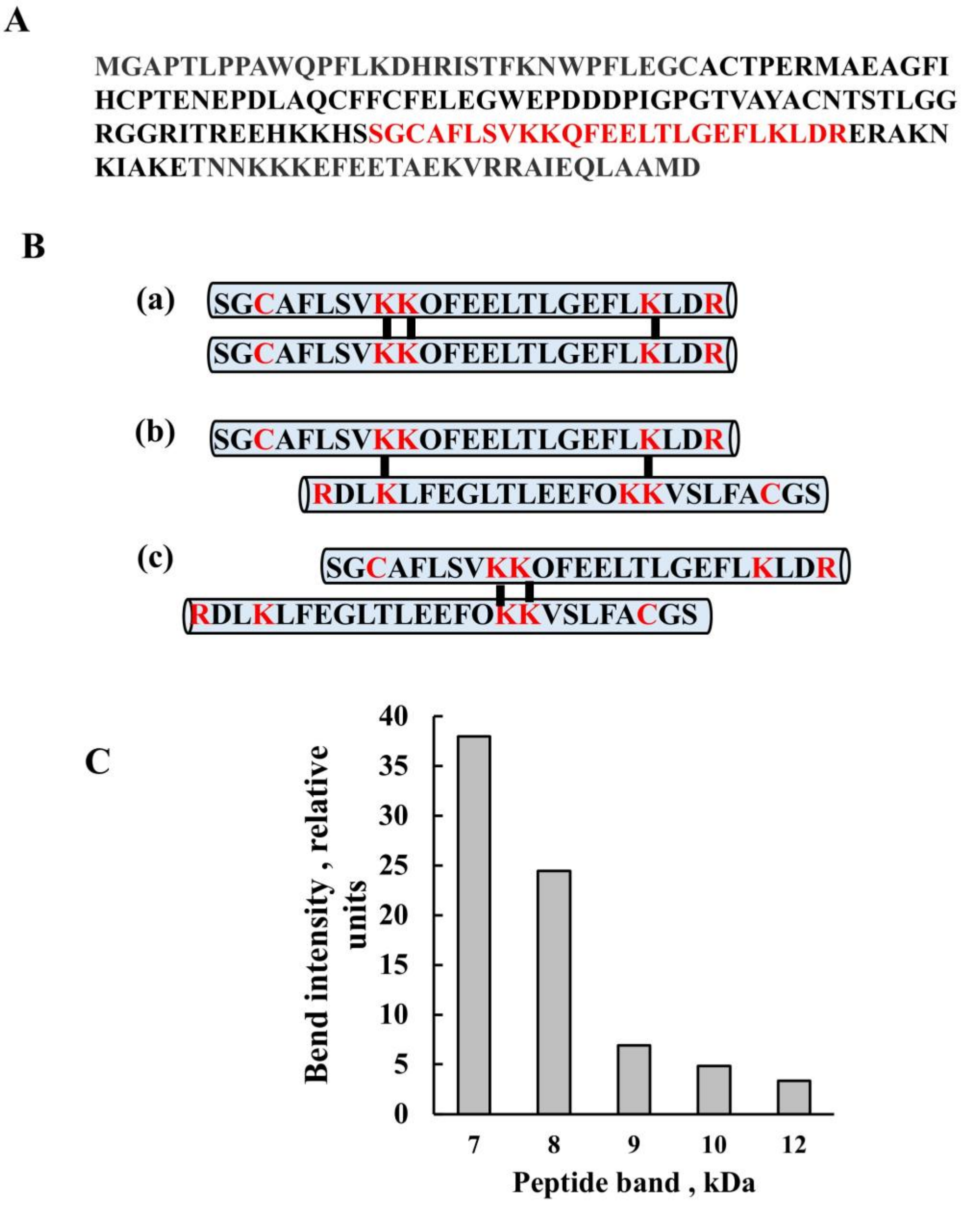

Fig. S2. Localization of the survivin dimerization domain, and peptide dimers/oligomer formation
(A) Localization of the peptide sequence within the survivin protein.
(B) Quantification of peptide bands, represented by their estimated molecular masses (kDa), corresponding to EGS crosslinked products shown in Fig. 1L.
(C) Predicted lysine residues within the peptide that may participate in EGS-mediated crosslinking, leading to the formation of distinct dimeric species.

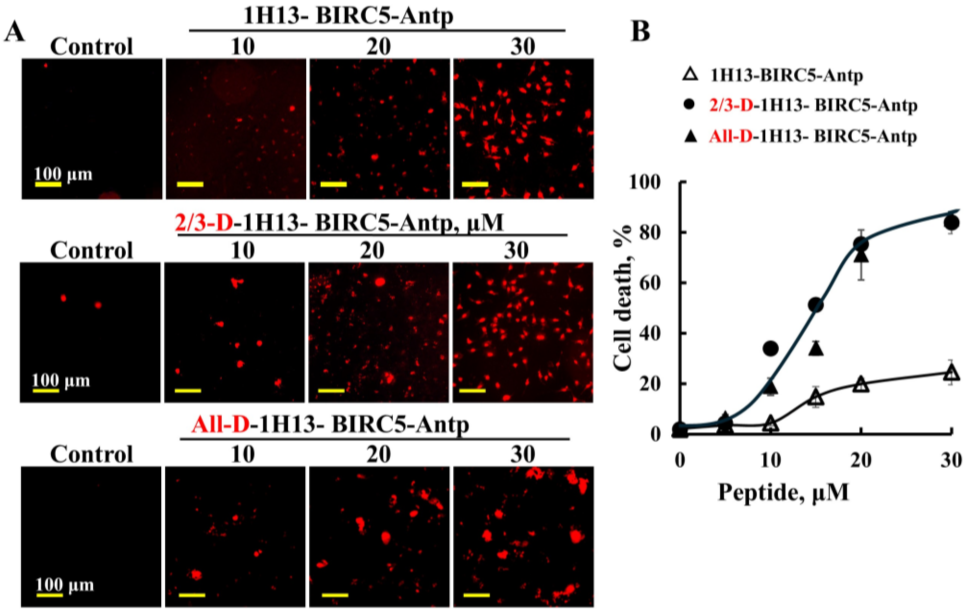

C

Peptide sequence	Targeted to	Cell death IC_{50}, µM (Maximal %)
SGCAFLSVKKQFEELTLGEFLKLD-R-[D-Arg]-RK	Nucleus<br>1H13-BIRC5	12<br>(95)
SGCAFLSVKKQFEELTLGEFLKLD-R-[D-Arg]-RK	2/3D-1H13-BIRC5	40<br>(95)
SGCAFLSVKKQFEELTLGEFLKLD-R-[D-Arg]-RK	All-D-1H13-BIRC	>>80<br>(25)
SGCAFLSVKKQFEELTLGEFLKLD-D-Arg–Dmt–Orn–Phe–NH_2	Mitochondria<br>1H13-BIRC5-Mito	40<br>(98)
SGCAFLSVKKQFEELTLGEFLKLD-D-Arg–Dmt–Lys–Phe–NH_2	2/3-D-1H13--BIRC5-Mito	25<br>(98)
RQIKIWFQNRRMKWKK-SGCAFLSVKKQFEELTLGEFLKLD	Cytosol<br>Antp-1H13--BIRC5	6<br>(95)
SGCAFLSVKKQFEELTLGEFLKLD-RQIKIWFQNRRMKWKK	1H13--BIRC5-Antp	6<br>(95)
SGCAFLSVKKQFEELTLGEFLKLD-RQIKIWFQNRRMKWKK	2/3-D-1H13--BIRC5-Antp	6<br>(95)
SGCAFLSVKKQFEELTLGEFLKLD-RQIKIWFQNRRMKWKK	All-D-1H13--BIRC5-Antp	>>30<br>(30)
SGCAFLSVKKQFEELTLGEFLKLDHRPYIAH	1H13-BIRC5-Tf	>>100<br>0
HRPYIAHSGCAFLSVKKQFEELTLGEFLKLD	Tf-1H13-BIRC5	>>100<br>0

Fig. S3. Cell death induction activity of the BIERC5/survivin-derived 1H13 peptide and its modified versions targeted to the cytosol, mitochondria, or nucleus

(A,B) A549 cells were incubated for 24h in serum-free growth medium with or without the indicated concentrations of 1H13-BIRC5-Antp or with D-amino-acid substitutions for the three amino acids in the C- or the N-terminus of the peptide (2/3D-1H13-BIRC5-Antp), or for all the amino acids in the peptide (All-D-1H13-BIRC5-Antp), and subjected to cell death analysis using PI staining and fluorescence imaging (A), and staining intensity was quantified (B). (C) Summary of the effects of the different versions of the peptide 1H13-BIRC5 on cell death. The cell-penetrating sequences appear in blue. The amino acids modified by replacing them with the D-confirmation of the amino acid are marked in red, and the targeted compartment is indicated. The concentrations required for 50% cell death induction (IC_{50}) and the maximal obtained cell death (%) are indicated. Results represent mean values ± SEM (n = 3).

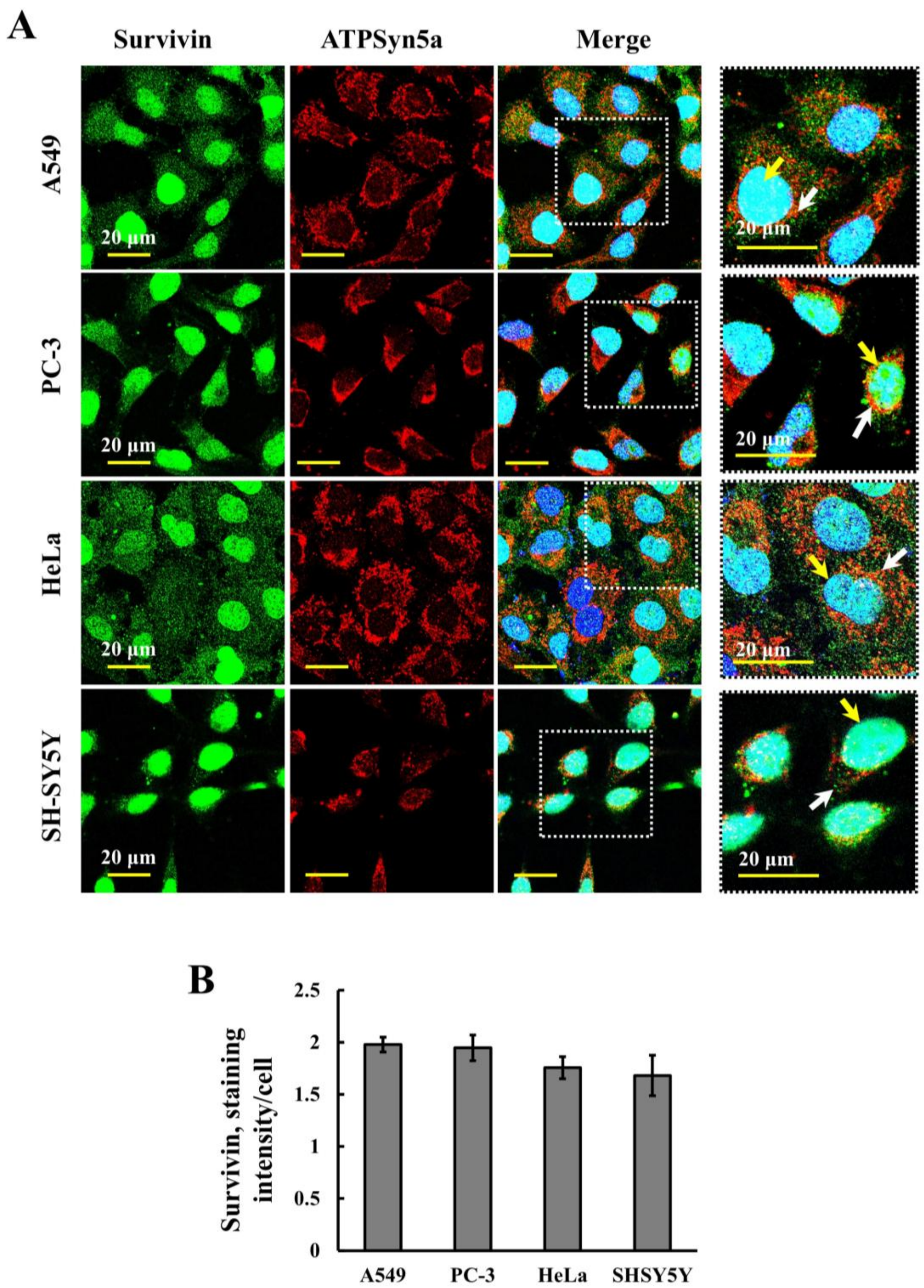

Fig. S4. Cellular localization and expression levels of survivin in different cell lines
(A) The indicated cell lines were grown on 13-mm glass coverslips, fixed, and subjected to co-immunofluorescence staining using anti-survivin and anti-ATP synthase 5a antibodies. Confocal images are shown, with yellow and white arrows marking nuclear and mitochondrial survivin, respectively. Enlarged views of selected regions are displayed on the right.
(B) Quantification of survivin fluorescence intensity per cell was performed using ImageJ (60–100 cells analyzed per sample), showing comparable survivin expression levels across all tested cell lines with no significant differences.

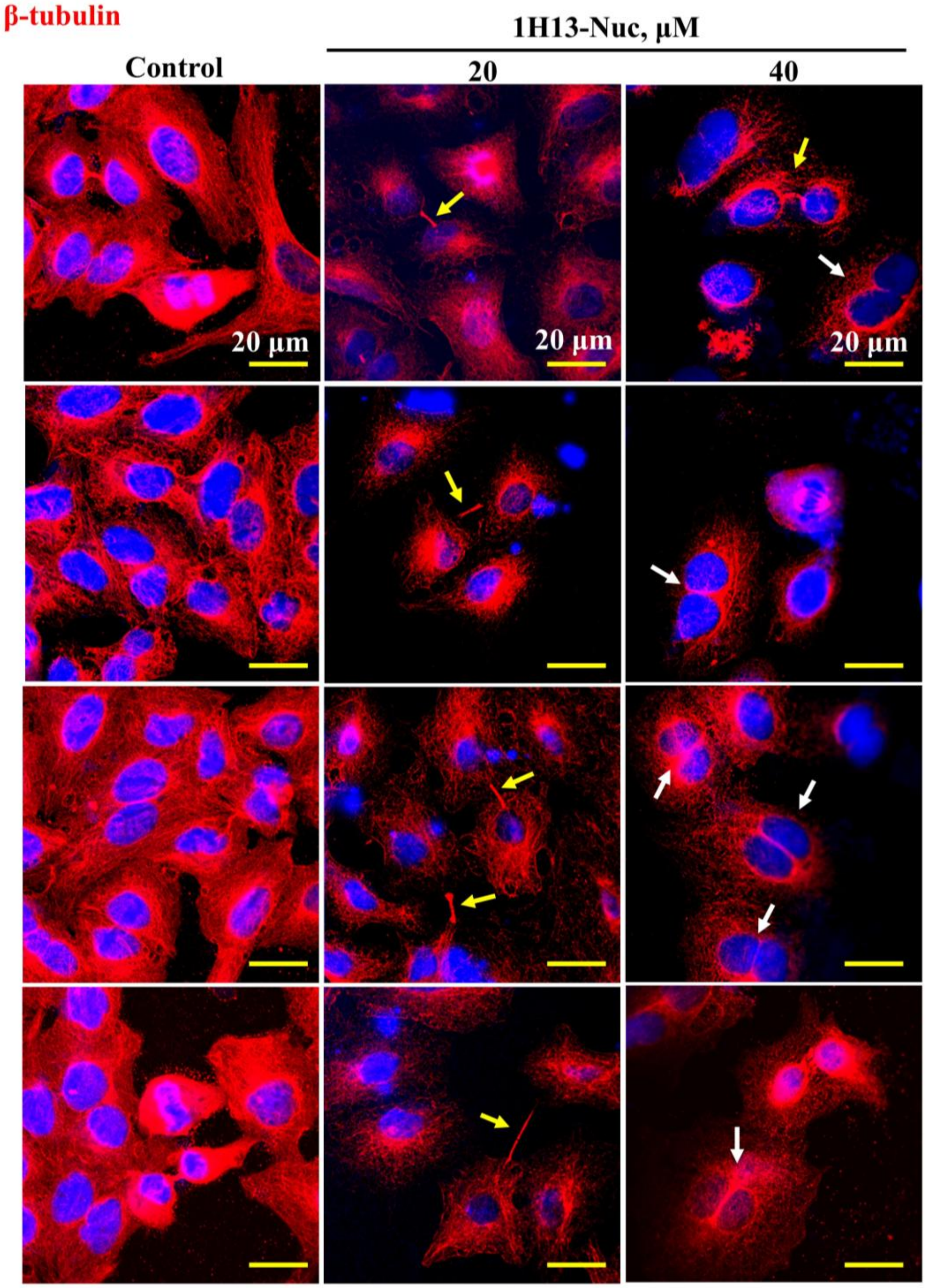

Fig. S5. 1H13-Nuc peptide decreased tubulin levels and impaired cell division in A549 cells

A549 cells were seeded on 13-mm glass coverslips, untreated (control) or treated with the 2/3D-1H13-Nuc peptide (20 or 40 μM, 24h), fixed, and subjected to IF using anti-β-tubulin antibodies. Several representative confocal images are shown for untreated and peptide-treated cells. Cells in late cytokinesis, where the microtubules between the daughter cells are visible, are indicated by yellow arrows. Bi-nuclear cells, likely resulting from incomplete cytokinesis, are indicated by white arrows.

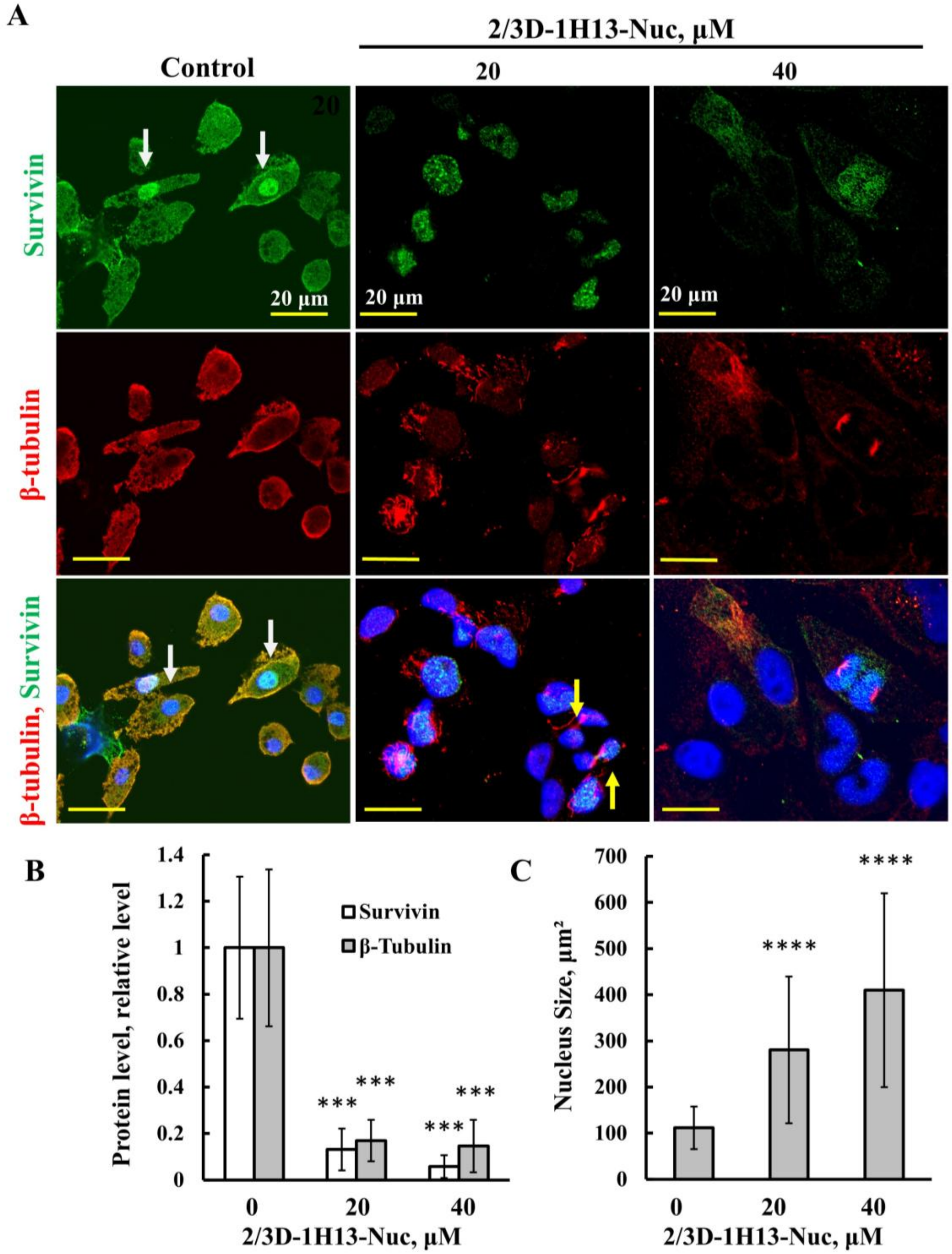

Fig. S6. 1H13-Nuc peptide decreased tubulin levels and impaired cell division in PC-3 cells (A) PC-3 cells were seeded on 13-mm glass coverslips, untreated (control) or treated with the 2/3D-1H13 peptide (20 or 40 μM, 24h), fixed, and subjected to co-IF using anti-survivin and anti-β-tubulin antibodies. Confocal microscope images are shown, with white arrows indicating nuclear survivin and yellow arrows incomplete cell division. (B) Quantification of survivin and tubulin levels per/cell in the IF-stained slides, using ImageJ (120–140 cells analyzed for each sample), showing a high decrease in survivin and tubulin levels. (C) Nuclear size was analyzed using ImageJ and is shown relative to its levels in untreated cells. Nucleus size was measured in control (n = 91), and peptide-treated 20 μM (n = 94) and 40 μM (n = 103) cells. Results represent means ± SEM (n = 3); *** $p < 0.001$;**** $p < 0.0001$.

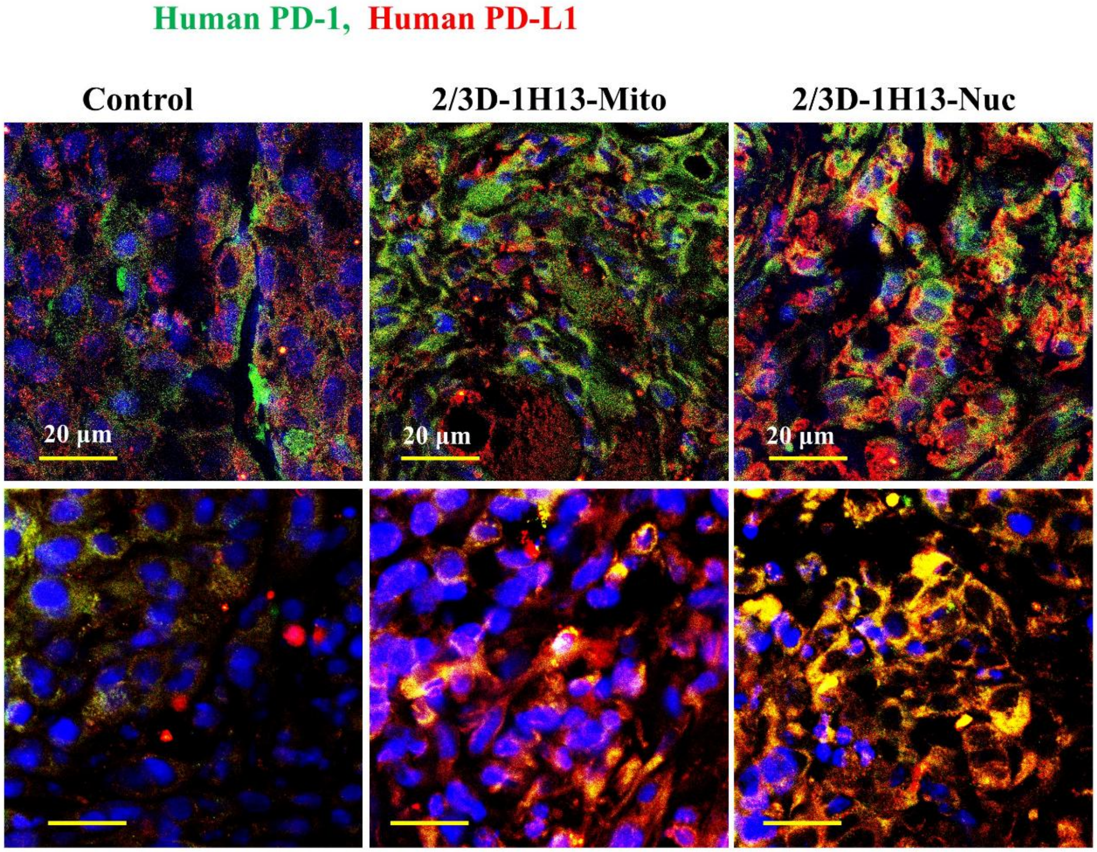

Fig. S7. 2/3D-1H13-peptide targeted to the nucleus or mitochondria increased PD-1 and PD-L1 expression in tumor cancer cells

Representative tumor sections from control mice and mice treated with the 2/3D-H13 peptide targeted to either the mitochondria or nucleus were co-IF stained with anti–PD-1 (human) and anti–PD-L1 (human) antibodies. Images from two tumors per group are shown.

References

1. Jeyaprakash, A.A., et al., *Structure of a Survivin-Borealin-INCENP core complex reveals how chromosomal passengers travel together.* Cell, 2007. **131**(2): p. 271-85.
2. Sasai, K., et al., *Aurora-C Interactions with Survivin and INCENP Reveal Shared and Distinct Features Compared with Aurora-B Chromosome Passenger Protein Complex.* PLoS One, 2016. **11**(6): p. e0157305.
3. Furuya, M., et al., *Interaction between survivin and aurora-B kinase plays an important role in survivin-mediated up-regulation of human telomerase reverse transcriptase expression.* International Journal of Oncology, 2009. **34**(4): p. 1061-1068.
4. Bolton, M.A., et al., *Aurora B kinase exists in a complex with survivin and INCENP and its kinase activity is stimulated by survivin binding and phosphorylation.* Mol Biol Cell, 2002. **13**(9): p. 3064-77.
5. Sarvagalla, S., et al., *Survivin - caspase protein-protein interaction: Experimental evidence and computational investigations to decipher the hotspot residues for drug targeting.* Journal of Molecular Structure, 2021. **1229**.
6. Dohi, T., et al., *An IAP-IAP complex inhibits apoptosis.* J Biol Chem, 2004. **279**(33): p. 34087-90.

7. Pohl, C. and S. Jentsch, *Final stages of cytokinesis and midbody ring formation are controlled by BRUCE.* Cell, 2008. **132**(5): p. 832-45.
8. Niu, T.K., et al., *Interaction of Beclin 1 with survivin regulates sensitivity of human glioma cells to TRAIL-induced apoptosis.* FEBS Lett, 2010. **584**(16): p. 3519-24.
9. Malcles, M.H., et al., *Characterisation of the anti-apoptotic function of survivin-DeltaEx3 during TNFalpha-mediated cell death.* Br J Cancer, 2007. **96**(11): p. 1659-66.
10. Wang, H., et al., *Acetylation directs survivin nuclear localization to repress STAT3 oncogenic activity.* J Biol Chem, 2010. **285**(46): p. 36129-37.
11. Dunajová, L., et al., *The N-terminus of survivin is a mitochondrial-targeting sequence and Src regulator.* Journal of Cell Science, 2016. **129**(14): p. 2707-2712.
12. Chang, W.H., et al., *Oncogenic RAS promotes MYC protein stability by upregulating the expression of the inhibitor of apoptosis protein family member Survivin.* J Biol Chem, 2023. **299**(2): p. 102842.
13. Feng, B.S., et al., *Survivin Impairs the Apoptotic Machinery in CD4+ T Cells of Patients with Ulcerative Colitis.* J Innate Immun, 2020. **12**(3): p. 226-234.
14. Suzuki, A., et al., *Survivin initiates cell cycle entry by the competitive interaction with Cdk4/p16(INK4a) and Cdk2/cyclin E complex activation.* Oncogene, 2000. **19**(29): p. 3225-34.
15. Wang, Q., A.M. Bode, and T. Zhang, *Targeting CDK1 in cancer: mechanisms and implications.* NPJ Precis Oncol, 2023. **7**(1): p. 58.
16. O'Connor, D.S., et al., *Regulation of apoptosis at cell division by p34cdc2 phosphorylation of survivin.* Proc Natl Acad Sci U S A, 2000. **97**(24): p. 13103-7.
17. Suzuki, A., et al., *Survivin initiates procaspase 3/p21 complex formation as a result of interaction with Cdk4 to resist Fas-mediated cell death.* Oncogene, 2000. **19**(10): p. 1346-53.
18. Park, S.H., et al., *An Inhibitor of the Interaction of Survivin with Smac in Mitochondria Promotes Apoptosis.* Chem Asian J, 2019. **14**(22): p. 4035-4041.
19. Verhagen, A.M., et al., *Identification of DIABLO, a mammalian protein that promotes apoptosis by binding to and antagonizing IAP proteins.* Cell, 2000. **102**(1): p. 43-53.
20. Song, Z.Y., X.B. Yao, and M. Wu, *Direct interaction between survivin and Smac/DIABLO is essential for the anti-apoptotic activity of survivin during taxol-induced apoptosis.* Journal of Biological Chemistry, 2003. **278**(25): p. 23130-23140.
21. Kim, J.Y., et al., *Nuclear interaction of Smac/DIABLO with Survivin at G2/M arrest prompts docetaxel-induced apoptosis in DU145 prostate cancer cells.* Biochemical and Biophysical Research Communications, 2006. **350**(4): p. 949-954.
22. Pandey, S., et al., *SMAC/Diablo controls proliferation of cancer cells by regulating phosphatidylethanolamine synthesis.* Mol Oncol, 2021.
23. Pandey, S.K., et al., *Non-apoptotic activity of the mitochondrial protein SMAC/Diablo in lung cancer: Novel target to disrupt survival, inflammation, and immunosuppression.* Front Oncol, 2022. **12**: p. 992260.
24. Paul, A., et al., *A New Role for the Mitochondrial Pro-apoptotic Protein SMAC/Diablo in Phospholipid Synthesis Associated with Tumorigenesis.* Mol Ther, 2018. **26**(3): p. 680-694.
25. Santhanam, M., et al., *Interaction of SMAC with a survivin-derived peptide alters essential cancer hallmarks: Tumor growth, inflammation, and immunosuppression.* Mol Ther, 2024. **32**(6): p. 1934-1955.
26. Babkoff, A., et al., *A direct interaction between survivin and myosin II is required for cytokinesis.* Journal of Cell Science, 2019. **132**(14).
27. Altieri, D.C., *The case for survivin as a regulator of microtubule dynamics and cell-death decisions.* Current Opinion in Cell Biology, 2006. **18**(6): p. 609-615.
28. Li, F., et al., *Control of apoptosis and mitotic spindle checkpoint by survivin.* Nature, 1998. **396**(6711): p. 580-4.
